# Biomolecular Complexation on the “Wrong Side”: A Case Study of the Influence of Salts and Sugars on the Interactions between Bovine Serum Albumin and Sodium Polystyrene Sulfonate

**DOI:** 10.1021/acs.biomac.2c00933

**Published:** 2022-09-22

**Authors:** Matjaž Simončič, Jozef Hritz, Miha Lukšič

**Affiliations:** † Faculty of Chemistry and Chemical Technology, University of Ljubljana, Večna Pot 113, SI-1000 Ljubljana, Slovenia; ‡ Central European Institute of Technology, Masaryk University, Kamenice 5, CZ-62500 Brno, Czechia; § Department of Chemistry, Faculty of Science, Masaryk University, Kamenice 5, CZ-62500 Brno, Czechia

## Abstract

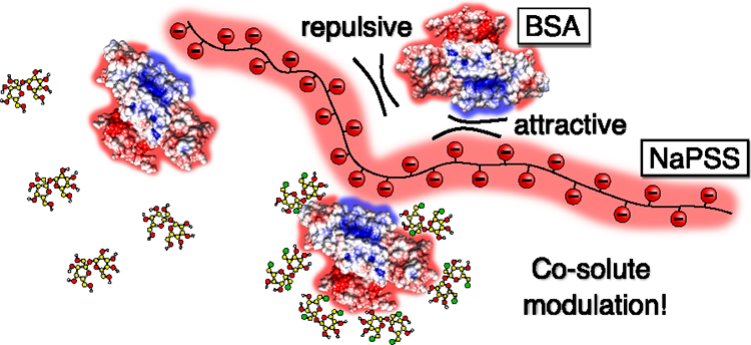

In the protein purification, drug delivery, food industry, and biotechnological applications involving protein–polyelectrolyte complexation, proper selection of co-solutes and solution conditions plays a crucial role. The onset of (bio)macromolecular complexation occurs even on the so-called “wrong side” of the protein isoionic point where both the protein and the polyelectrolyte are net like-charged. To gain mechanistic insights into the modulatory role of salts (NaCl, NaBr, and NaI) and sugars (sucrose and sucralose) in protein–polyelectrolyte complexation under such conditions, interaction between bovine serum albumin (BSA) and sodium polystyrene sulfonate (NaPSS) at pH = 8.0 was studied by a combination of isothermal titration calorimetry, fluorescence spectroscopy, circular dichroism, and thermodynamic modeling. The BSA–NaPSS complexation proceeds by two binding processes (first, formation of intrapolymer complexes and then formation of interpolymer complexes), both driven by favorable electrostatic interactions between the negatively charged sulfonic groups (−SO_3_^–^) of NaPSS and positively charged patches on the BSA surface. Two such positive patches were identified, each responsible for one of the two binding processes. The presence of salts screened both short-range attractive and long-range repulsive electrostatic interactions between both macromolecules, resulting in a nonmonotonic dependence of the binding affinity on the total ionic strength for both binding processes. In addition, distinct anion-specific effects were observed (NaCl < NaBr < NaI). The effect of sugars was less pronounced: sucrose had no effect on the complexation, but its chlorinated analogue, sucralose, promoted it slightly due to the screening of long-range repulsive electrostatic interactions between BSA and NaPSS. Although short-range non-electrostatic interactions are frequently mentioned in the literature in relation to BSA or NaPSS, we found that the main driving force of complexation on the “wrong side” are electrostatic interactions.

## Introduction

Protein–polyelectrolyte (PE) complexation depends on numerous parameters, such as the charge stoichiometry of the components, the charge density of the PE, the charge anisotropy of the protein surface, the mixing order of the components, the temperature, pH and ionic strength of the medium, and the presence of co-solutes, to name a few. Protein–PE complexation usually occurs in the presence of co-solutes such as salt ions, buffer species, and so forth. For this reason, the study of the influence of other components on complexation is essential, especially since protein–PE complexation is used in various applications, such as solubilization of components in the food industry,^1,2^ drug delivery,^3^ and protein purification,^4−6^ which are often at least ternary systems. Protein–PE complexation is not limited to biotechnological applications, as complexation also occurs in cellular processes, for example, in the formation of membrane-less organelles,^7,8^ complexation between proteins and nucleic acids,^9−11^ and so forth. For a more comprehensive overview, the reader is referred to reviews on protein–PE complexation.^12−16^

The interaction between an oppositely charged protein and a PE leads to the formation of a protein–PE complex. However, complexation is not limited to proteins and PEs carrying opposite charges, as it can also occur on the “wrong side” of the protein isoionic point, that is, when both molecules carry the same net charge.^17−20^ The origin of this well-established phenomenon is still debated. In a purely electrostatic framework, it can be explained by two different mechanisms: the charge-patch^21−24^ and the charge-regulation mechanism.^20,25,26^ The attraction between the charge patches, which arise due to the heterogeneous charge distribution on the protein surface, and the PE can locally overcome the overall repulsive charge–charge interactions between the two like-charged macromolecules. The charge-regulation mechanism, on the other hand, suggests that the presence of a highly charged molecule near the protein surface can alter the variable charge of weak acidic and basic amino acid residues, allowing the formation of strong attractive interactions. A plausible explanation is also the presence of short-range non-electrostatic interactions (commonly referred to as “hydrophobic interactions”);^22,27,28^ however, such an explanation is somewhat controversial and has been debated in the literature.^17,21,29,30^

**Figure 1:**
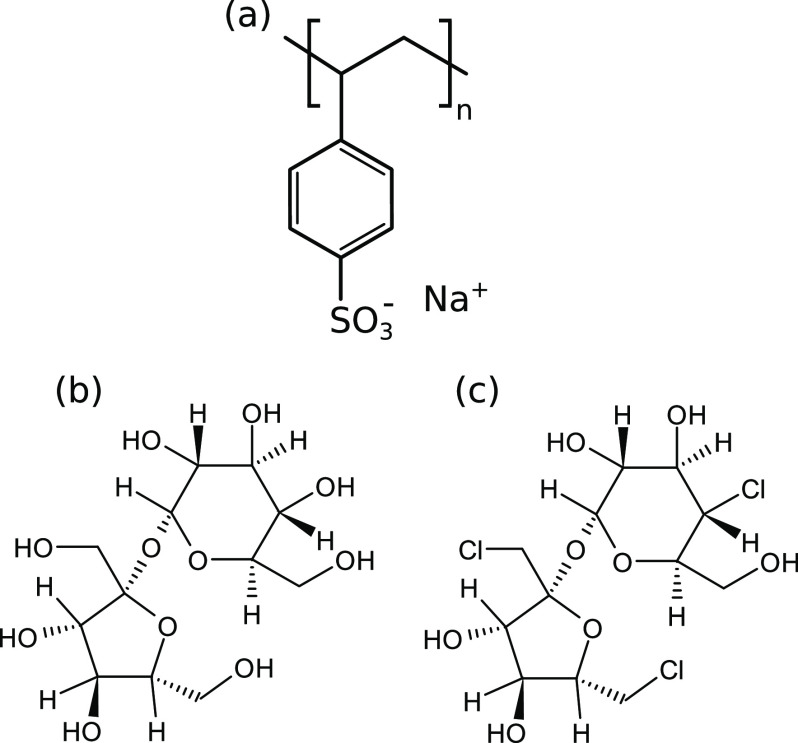
Chemical structures of (a) repeating unit of NaPSS, (b) sucrose, and (c) sucralose.

In the present study, we focus on the effect of co-solutes on the complexation between bovine serum albumin (BSA) and sodium polystyrene sulfonate (NaPSS; Figure 1a) at pH = 8.0, which is above the isoionic point of BSA (*pI*_BSA_ ≈ 4.7^31^). The issue correlates strongly with our previous work,^32^ in which the effect of co-solutes was evaluated in terms of their ability to hinder or promote phase separation in the BSA/NaPSS system as the pH of the solution was varied. Here, we focus on how complexation between BSA and NaPSS is affected at the molecular level by two types of co-solutes: salts (NaCl, NaBr, and NaI) and sugars (sucrose and sucralose; Figure 1b,c).

Isothermal titration calorimetry (ITC) is a technique often employed for evaluating complexation between proteins and PEs. Thermodynamic analysis of binding profiles provides useful data on the mechanism of protein–PE complexation and has been used extensively in this field. Calorimetric studies of complex formation between proteins and synthetic PEs, such as between BSA and polyacrylamide gel electrophoresis (PAGE)-based PEs,^33^ human serum albumin (HSA) and polyacrylic acid,^34^ and natural PEs, for example, BSA and gum arabic,^35^ lysozyme and heparin,^36^ β-lactoglobulin (BLG) and acacia gum,^37^ BLG and pectin,^38^ and so forth, have been conducted. Extensive studies have also been carried out on the complexation of proteins with spherical PE brushes.^4,39−41^ From a thermodynamic point of view, complex formation between a protein and PE can be entropically driven, as counterions (or water molecules) are released from the interface between the protein and PE upon association, or enthalpically driven if favorable charge–charge interactions are involved. The presence of co-solutes modulates the driving forces involved in complexation and influences the mechanism of association, which can be monitored by ITC.

Since complexation between proteins and PEs is usually dominated by electrostatic interactions, the presence of salts should have the greatest influence on the process. The modulation is caused by the screening of electrostatic interactions, which can be attractive or repulsive and depend mainly on pH and the ionic strength of the medium as well as on the charge density of the PE and the charge anisotropy on the protein surface. Evaluating the association as a function of ionic strength provides insights into the electrostatic forces driving the complexation. A nonmonotonic dependence^5,21,22,34,42^ of the binding constant on ionic strength, as opposed to a monotonic one,^21,43^ usually indicates the presence of two different types of interactions that exhibit an opposite dependence on ionic strength. However, the effect depends on the charge of the protein, which is predominantly regulated by pH of the medium, as seen in the BLG/NaPSS system (see ref (21)). Moreover, the modulatory effect of salts is not only dependent on ionic strength, as it often also depends on the chemical identity of the added salt ions^32,44^ and on the protein/PE system itself.^17,21,29,30,33^

The stabilizing effect of sugars is usually associated with their water-structuring abilities, leading to the so-called preferential hydration phenomenon, in which sugars are excluded from the protein interface rather than interacting directly with the amino acid residues of the protein. Consequently, the effect of sugars on protein–PE complexation is much less documented,^45^ especially since their modulatory role is less obvious in electrostatically dominated systems. However, in our recent work, we have shown that the chemically modified sugar sucralose (the chlorinated analogue of sucrose) can affect protein–PE complexation and prevent the onset of complex formation in the BSA/NaPSS system around the isoionic point of BSA.^32^ In this work, we focus on the influence of the two types of co-solutes (salts, sugars) on the complexation between BSA and NaPSS. We also present mechanistic explanations for the modulatory effects.

## Experimental Section

### Materials

BSA (fatty acid-free; LOT number: SLCB1005), NaPSS (average molecular weight 70,000 g/mol), sucrose, sucralose, and NaI (>99%) were purchased from Sigma-Aldrich. Sodium salts of >99% purity (NaCl, NaBr, Na_2_HPO_4_·2H_2_O) and 1 mol/L NaOH solution were purchased from Merck KGaA.

### Preparation of Buffer, Protein, and PE Stock Solutions

Phosphate buffer solution with a concentration of 7.3 mM was prepared by dissolving the appropriate amount of Na_2_HPO_4_·2H_2_O in Milli-*Q* water and titrating with 1 M (mol/L) sodium hydroxide solution to pH = 8.0. The ionic strength of such a buffer (*I*_buffer_) is at 25 °C equal to 20 mM. In cases where the buffer also contained a co-solute (salts, sugars), the buffer-(co-solute) solution was prepared by dissolving the co-solute along with Na_2_HPO_4_·2H_2_O. In the presence of sugars or the absence of a salt, *I*_total_ is equal to *I*_buffer_. In the presence of salts, *I*_total_ is the sum of the ionic strength of the buffer (*I*_buffer_) and the added salt (*I*_salt_), which is for monovalent salts equal to their molar concentration.

The pH of solutions was measured using the Iskra pH meter (Iskra, Slovenia) and a combined glass micro-electrode InLab Micro (Mettler Toledo, Switzerland).

NaPSS was dissolved in Milli-*Q* water and was purified by dialysis of the aqueous solution against Milli-*Q* water until the conductivity of the dyalizate matched that of water (less than 2 μS/cm). For this purpose, dialysis tubing cellulose membranes (Sigma-Aldrich; MW cut-off: 14,000 g/mol) were used. The dialyzed solution was freeze-dried by using the HETOS-ICC freeze dryer (CD 52-1), and the dry NaPSS was stored for further use.

The stock solution of BSA was prepared by dissolving the powder in the appropriate buffer-(co-solute) solution (pH = 8.0), and the solution was then dialyzed against the corresponding buffer for 24 h with fresh buffer changes every 8 h. Spectra/Por membranes (MW cut-off: 3,500 g/mol) were used for the purification step. The stock solution was filtered through a 0.45 μm filter (Minisart, Sartorius) and stored for further use.

NaPSS stock solutions were prepared by dissolving the lyophilized powder in the same buffer-(co-solute) solution as that used for the protein. The concentration of the protein in the stock solution was determined using the NanoDrop OneC Microvolume UV–Vis spectrophotometer (Thermo Scientific, USA) at 280 nm (ε = 0.667 L g^–1^ cm^–1^ at 25 °C^46^). The concentration of NaPSS was determined using a Cary 100 Bio (Varian, USA) spectrophotometer at 262 nm (ε = 1.82 L g^–1^ cm^–1^ at 25 °C^47^). In this work, the concentration of NaPSS is given as the concentration of moles per liter of solution by assuming that the number of repeating units of NaPSS in a molecule is equal to 340. Unless stated otherwise, the NaPSS to BSA concentration ratio is defined as *r* = [NaPSS]/[BSA], where [NaPSS] and [BSA] denote the molar concentrations of NaPSS and BSA, respectively.

The preparation of working solutions is described in the following sections.

### Isothermal Titration Calorimetry

ITC experiments were performed using MicroCal Auto-iTC200 (Malvern Panalytical). In most cases, the NaPSS-containing solution was added to a BSA-containing solution (we refer to this experiment as NaPSS-to-BSA titration). The sample cell initially contained a 50 μM BSA solution and was titrated with a 56 μM solution of NaPSS. Both components were dissolved in the appropriate buffer-(co-solute) solution. A total of 30 consecutive injections, divided into twenty 1 μL and ten 2 μL injections, were performed to obtain sufficient data points to characterize the two-stage process in a single titration experiment. The heat effect of reference titrations (see Figure S1 in the Supporting Information file) was subtracted. The stirring rate was set to 500 RPM. All measurements were performed at 25 °C.

In the reverse titration, a BSA-containing solution was added to a NaPSS-containing solution (we refer to this experiment as BSA-to-NaPSS titration). In this case, the sample cell initially contained 5.6 μM NaPSS solution and was titrated with a 200 μM BSA solution. All other parameters were the same as those for the NaPSS-to-BSA titration. With these concentrations, we were able to record the entire binding isotherm using the same ITC protocol as that used for the NaPSS-to-BSA titration.

### Model analysis

The integration of the ITC thermogram to obtain the binding isotherm was done using NITPIC software.^48^ A “two sets of independent sites” (TSIS) binding model^49,50^ was used to fit the experimental binding isotherms. The model is based on the Langmuir isotherm, which considers the binding process an equilibrium between the empty and occupied adsorption sites of the macromolecule and the number of macromolecules in the solution. The apparent intrinsic binding (equilibrium) constants, *K*_b,*i*_, for both sets (*i* = 1 or 2) can be expressed as
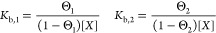
1where Θ_1_ and Θ_2_ are fractions of sites occupied by ligand *X* and [*X*] denotes the molar concentration of the free ligand. The total concentration of the ligand, *X*_t_, in the active volume, *V*_0_, of the solution can be expressed as
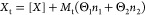
2where *M*_t_ is the bulk concentration of the macromolecule in the active volume and *n*_1_ and *n*_2_ designate the numbers of binding sites of each set (binding stoichiometries). By combining eqs 1 and 2, we obtain a cubic equation which can be solved numerically. Fitting of the experimental data was done using Microcal ORIGIN software available with the ITC instrument. The heat release, Δ*H*, upon ligand injection can be calculated as
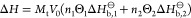
3where *H*_b,1_^⊖^ and Δ*H*_b,2_^⊖^ denote the changes in the standard binding enthalpy for both processes. The results of the fit are six parameters, corresponding to a reference temperature of the experiment: *K*_b,1_, *K*_b,2_, *n*_1_, *n*_2_, *H*_b,1_^⊖^, and Δ*H*_b,2_^⊖^.

Furthermore, the change in the standard binding free energy, Δ*G*_b,*i*_^⊖^, can be calculated from the thermodynamic relation
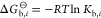
4where *R* is the gas constant and *T* is the absolute temperature of the solution. The change of standard binding entropy, Δ*S*_b,*i*_^⊖^, can be obtained from the definition
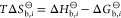
5

ITC measurements were performed in duplicate. Global fitting of the TSIS model was performed on the two replicate titration experiments. The least-squares Levenberg–Marquardt algorithm was used, and uncertainties in the fitting parameters were determined from the variance–covariance matrix. The standard state corresponds to the concentration *c*^⊖^ = 1 mol/L.

### Fluorimetry

Fluorimetric measurements were performed using an LS 55 PerkinElmer (USA) fluorimeter. Unless stated otherwise, the temperature was kept constant at 25 °C with the temperature being regulated using a PerkinElmer PTP-1 Peltier system. The excitation wavelength was set to 295 nm (selective excitation of tryptophan residues), and the emission scan range was from 300 to 450 nm. The bandwidth for the excitation and emission slits was 5 nm. The emission spectrum of 2 μM BSA in a cuvette with an optical path length of 1 cm in the absence and presence of NaPSS was measured. All solutions were prepared in phosphate buffers (pH = 8.0) with varying ionic strengths, vortexed for ∼10 s, and left to equilibrate at room temperature for 15 min before the measurement. To be consistent with our previous measurements,^32^ the excitation wavelength for BSA–sugar solutions was set to 280 nm, and emission spectra of 0.5 μM BSA were recorded between 300 and 400 nm. Solutions for those measurements were prepared in a phosphate buffer with an ionic strength of 100 mM (*c* = 36 mM, pH = 8.0). In all cases, the references were subtracted.

The interaction between the fluorophore and the quencher (NaPSS, co-solute) can be described using the well-known Stern–Volmer equation^51^
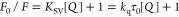
6where *F*_0_ and *F* represent the fluorescence intensities (recorded at 350 nm for λ_ex_ = 295 nm and 347 nm for λ_ex_ = 280 nm) in the absence and presence of the quencher, respectively. *K*_SV_ is the Stern–Volmer quenching constant, [*Q*] is the molar concentration of the quencher, *k*_q_ is the bimolecular quenching rate constant, and τ_0_ is the average lifetime of the fluorophore in the absence of the quencher, which is for tryptophan in BSA reported to be 5.8–6.0 ns.^52,53^ Fluorescence intensities were corrected for the inner-filter effect using the Lakowicz model:^51^, where *F*_corr_ and *F*_obs_ are the corrected and measured (uncorrected) fluorescence intensities at the emission wavelength, *A*_ex_ and *A*_em_ are the absorbance values at the excitation and emission wavelength, respectively.

The uncertainty of the parameters was determined from the least-squares fit (Levenberg–Marquardt algorithm). All measurements were done in duplicate with the results being reported as the average of the two measurements.

### Circular Dichroism

Far-UV range CD measurements (200–260 nm) were performed to assess changes in the secondary structure of BSA upon complexation with NaPSS in the absence and presence of co-solutes (NaCl, sucrose, sucralose). The temperature was regulated using a Julabo F25-ME thermostat and was set to 25 °C. The concentration of BSA was for all solutions 2.5 μM. Phosphate buffer was used as the solvent (pH = 8.0). Measurements were carried out on a Jasco-1500 CD spectrometer using quartz cuvettes (optic path length of 0.1 cm). Raw CD spectra were normalized on the concentration and optic path length and are presented as mean residue ellipticity ([θ]), calculated as
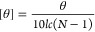
7where the θ, *l*, *c*, and *N* represent the measured ellipticity in mili-degrees, the optical path length in cm, the molar concentration of BSA in mol/L, and the number of amino acid residues of BSA, respectively. All measurements were done in duplicate with the resulting CD spectra presented as the average of two measurements. In all cases, the references were subtracted. Conformational changes of BSA were estimated by using the online server BeStSel.^54^

### UV–Vis Measurements

UV–vis absorption spectra were recorded on a Cary 100 Bio spectrophotometer (Varian) at 25 °C. A Peltier block was used for temperature regulation, along with a Cary temperature controller (Agilent) pre-thermostat. Quartz cuvettes with a 1 cm optical path were used to measure the spectra of 9 μM BSA in the presence and absence of NaPSS. Phosphate buffer was used to prepare all solutions (pH = 8.0, *I*_total_ = 20 mM). In all cases, the references were subtracted.

### Visualization of the Molecular Surface

For the assessment of the charge distribution and the hydrophobicity of the protein surface, the structure of BSA was taken from the Protein Data Bank (PDB ID: 4f5s).^55^

The protonation states of amino acid residues at pH = 8.0 were determined with PDB2PQR, an online tool for the setup of Poisson–Boltzmann electrostatic calculations,^56^ using the PARSE force field. The electrostatic potential on the protein surface (solvent-excluded surface area) was calculated by solving the linear Poisson–Boltzmann equation using the DelPhiPKa web server^57^ and then visualized using Chimera software.^58^ The probe radius was set at 0.14 nm, the internal and external dielectric constants were 4 and 78, respectively, and the temperature was set at 25 °C. The electrostatic potentials at different ionic strengths were determined considering different salt concentrations.

The hydrophobicity of the protein surface was visualized using the Kyte and Doolittle hydropathy scale.^59^ Each amino acid residue was assigned a color according to its hydropathy index on the scale from the most hydrophilic (arginine; −4.5), colored blue, to the most hydrophobic (isoleucine: 4.5), colored orange. The molecular surface (solvent-excluded surface area) was colored according to the color codes for each residue. Visualization was performed using the Chimera package.^58^

## Results and Discussion

As the reference measurements for the BSA–NaPSS complexation on the “wrong side” (pH ≫ *pI*_BSA_), we consider protein–PE solutions without added co-solutes (i.e., salt- and sugar-free solutions). These findings are used later to explain the effect of salts (NaCl, NaBr, NaI) and sugars (sucrose, sucralose) on BSA–NaPSS complexation.

### BSA–NaPSS Complexation without the Co-Solute Present

The complexation between BSA and NaPSS at pH = 8.0 occurs above the isoionic point of the protein (*pI*_BSA_ ≈ 4.7^31^). Since under these conditions, the net charge of the protein has the same sign as that of the PE (NaPSS), the phenomenon is referred to as complexation on the “wrong side”.^17−19^ In general, the local attractive interactions must overcome the overall repulsive interactions caused by the electrostatic repulsion between the like-charged protein and PE for complex formation to occur.

**Figure 2:**
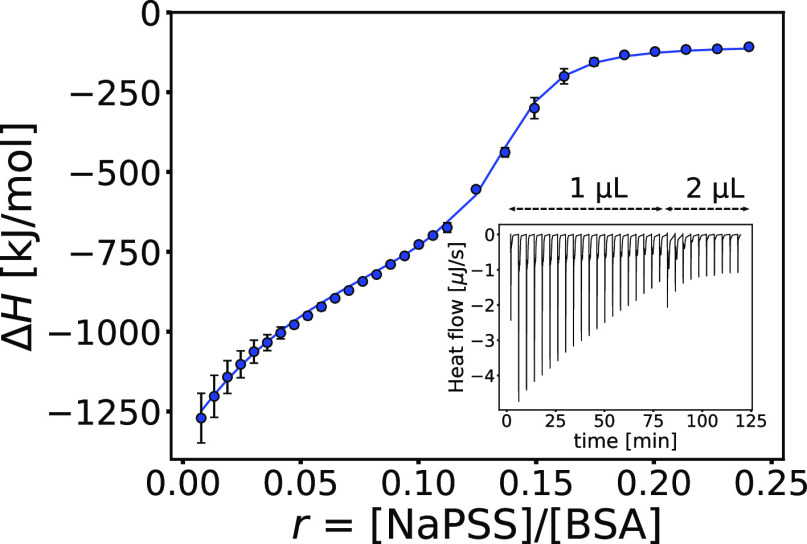
The complexation on the “wrong side” between BSA and NaPSS proceeds in two tightly coupled binding processes. The binding isotherm (blue circles) for the NaPSS-to-BSA titration, obtained by integrating the thermogram (inset). The fit of a TSIS binding model is shown as a solid blue line. All solutions were prepared in phosphate buffer (*I*_total_ = 20 mM, pH = 8.0), and data were collected at 25 °C.

First, the heat effects of titrating a BSA solution (solution in the calorimetric cell) with a NaPSS solution (solution in the syringe) without any addition of the co-solute are investigated. Both macromolecules were dissolved in aqueous phosphate buffer (*I*_total_ = 20 mM, pH = 8.0). Figure 2 shows the binding isotherm along with the corresponding thermogram (inset) for the NaPSS-to-BSA titration. The binding isotherm was obtained by integrating a sequence of strongly exothermic peaks. Two tightly coupled binding processes can be deduced from the shape of the isotherm. We used a so-called TSIS binding model to fit the experimental binding isotherm (see the section Model Analysis). Since the focus of this work is on the modulating effect of co-solutes on BSA–NaPSS complexation, we selected the simplest model capable of describing the binding isotherms indicative of two binding processes.

Considering that protein–PE complexation is often associated with the self-assembly of both macromolecules into larger structures, the two above-mentioned binding processes might signify two structuring events.^37,38^ The reasoning follows from Tainaka’s theory,^60^ according to which, complexation is initiated by the formation of intrapolymer complexes (we name this the first binding process), followed by their association into larger interpolymer complexes (named in this work the second binding process). No insoluble BSA–NaPSS complexes were observed at pH = 8.0. However, at lower pH values, the latter process may lead to phase separation.^32^ Complex formation is initiated by the attachment of smaller protein molecules to longer NaPSS chains, forming a pearl-necklace-shaped structure.^61^ The association of such intrapolymer complexes into larger associates proceeds almost simultaneously, and a clear separation between the structuring regimes (binding processes) is difficult to determine using ITC. According to the shape of the binding isotherm shown in Figure 2, the boundary between the two binding processes could be somewhere between *r* ≈ 0.05 and 0.10. The formation of interpolymer complexes requires that BSA be able to bind to multiple NaPSS molecules, that is, it should have at least two or more binding domains (patches). The electrostatic potential map of the BSA surface at pH = 8.0 and at an ionic strength of 20 mM is shown in Figure S2 (left). The map clearly shows two larger patches of positive charge (labeled A and B) that could facilitate binding with the negatively charged polyanion at pH = 8.0. Similarly, two positive patches in approximately same locations were identified by Grymonpré et al.^24^ for the binding of the structurally similar HSA to hyaluronic acid under conditions corresponding to complexation on the “wrong side”.

Attractive electrostatic interactions between the positively charged patches of the BSA and the negatively charged NaPSS may be responsible for both the complexation on the “wrong side” and for the two-process character of BSA–NaPSS binding. An alternative mechanism to the charge-patch mechanism used in the literature to explain the complexation on the “wrong side” is the so-called charge-regulation mechanism. Although the two mechanisms (charge-patch and charge-regulation) are not mutually exclusive, as recently shown by Lunkand et al.,^62^ the ability of BSA to charge-regulate at pH ≈ 8.0 is low.^25^ In addition, charge regulation is usually considered at low ionic strengths (a few mM), and bearing in mind that we performed our measurements at larger ionic strengths (*I*_total_ = 20 mM), the association between NaPSS and BSA at pH = 8.0, where BSA also has a large dipole moment,^63,64^ is most likely a consequence of the charge-patch mechanism.

Thermodynamic analysis of complex formation can provide valuable insights into the complexation mechanism. The complexation can be enthalpically driven as a consequence of favorable charge–charge interactions or entropically driven due to the release of condensed counterions (or water molecules) upon association between the protein and PE. The parameters obtained by fitting the NaPSS-to-BSA binding isotherm (Figure 2) with the TSIS model  are given along with Δ*G*_b,*i*_^⊖^ and *T*Δ*S*_b,*i*_^⊖^ (eqs 4 and 5) in Table 1. We note that the absence of a plateau on the binding isotherm at low molar ratios (see Figure 2, *r* → 0) did not allow an unambiguous determination of the binding stoichiometry (*n*_1_) and the change in the standard binding enthalpy (Δ*H*_b,1_^⊖^) of the first binding process; therefore, only the apparent binding constant (*K*_b,1_) and the corresponding standard free energy change (*G*Δ_*b*,1_^⊖^) for this process are given in Table 1. For the second binding process, all three fitting parameters (*K*_b,2_, Δ*H*_b,2_^⊖^, and *n*_2_) could be obtained.

**Table 1:** Thermodynamic Parameters of the First and Second Binding Processes Obtained by Fitting the TSIS Model to the Binding Isotherm of the NaPSS-to-BSA and BSA-to-NaPSS Titration^a^

	I. binding process		II. binding process				
order of mixing	*K*_b,1_ × 10^–7^	Δ*G*_b,1_^⊖^ [kJ/mol]	*K*_b,2_ × 10^–7^	Δ*G*_b,2_^⊖^ [kJ/mol]	*n*_2_	Δ*H*_b,2_^⊖^ [kJ/mol]	*T*Δ*S*_b,2_^⊖^ [kJ/mol]
NaPSS-to-BSA	4 ± 1	–43.4 ± 0.6	2 ± 1	–42 ± 2	0.12 ± 0.02	(−4 ± 2) × 10^2^	(−4 ± 2) × 10^2^
BSA-to-NaPSS	9 ± 4	–45 ± 1	0.13 ± 0.01	–34.9 ± 0.2	3.68 ± 0.06	–43.7 ± 0.6	–8.8 ± 0.7

a All solutions were prepared in the phosphate buffer (*I*_total_ = 20 mM, pH = 8.0). Data were collected at *T* = 25 °C. Apparent binding constants of the first and second binding processes (*K*_b,1_, *K*_b,2_), binding stoichiometry (*n*_2_), and change in the standard binding enthalpy (Δ*H*_b,2_^⊖^) of the second binding process are fitting parameters, while the corresponding binding free energy changes (Δ*G*_b,1_^⊖^, Δ*G*_b,2_^⊖^) and change in standard entropy (*T*Δ*S*_b,2_^⊖^) were calculated via eqs 4 and 5, respectively.

The binding constants of the first process (formation of intrapolymer complexes), *K*_b,1_ = (4 ± 1) × 10^7^, and of the second process (formation of interpolymer complexes), *K*_b,2_ = (2 ± 1) × 10^7^, are of the same order of magnitude. The binding constant *K*_b,1_ is slightly larger than *K*_b,2_. Since a reliable value for the binding enthalpy change of the first process (Δ*H*_b,1_^⊖^) could not be determined, we cannot conclude whether the main contribution to the binding free energy of the first process, Δ*G*_b,1_^⊖^ = (−43.4 ± 0.6) kJ/mol, is enthalpic or entropic. Nevertheless, we can see from the binding isotherm (Figure 2) that *H*_b,1_^⊖^ < 0, indicating the presence of favorable electrostatic interactions between positive patches of BSA and the negatively charged −SO_3_^–^ groups of NaPSS.

In the case of the second binding process, the enthalpic, Δ*H*_b,2_^⊖^ = (−4 ± 2) × 10^2^ kJ/mol, and entropic, *T*Δ*S*_b,2_^⊖^ = (−4 ± 2) × 10^2^ kJ/mol, contributions to the binding free energy, Δ*G*_b,2_^⊖^, are both negative, of comparable magnitudes, and accompanied with quite large uncertainties. Δ*H*_b,2_^⊖^ < 0 can be mainly attributed to favorable electrostatic interactions, while the unfavorable entropic contribution (*T*Δ*S*_b,2_^⊖^ < 0) arises from a large decrease in the configurational entropy of long NaPSS molecules upon association with BSA. The counter-ion release upon complexation [which would result in favorable (positive) entropy change] is most likely overshadowed by the loss in configurational entropy of the PE. It can be concluded that favorable electrostatic interactions are the main driving forces of the second binding process.

Since BSA was titrated with NaPSS, the inverse value of the binding stoichiometry of the second binding process, 1/*n*_2_ (*n*_2_ = 0.12 ± 0.02), provides the information that on average, 7–10 BSA molecules are bound to one NaPSS chain during the formation of interpolymer complexes under conditions studied. This estimate includes the protein molecules which were bound during the first binding process, for which the unbiased value of *n*_1_ could not be determined.

The validity of the TSIS model was tested by applying the model to complementary fluorimetry titration data (see Figure S3 in the Supporting Information file). The binding constants for the first and second processes are given in Table S1. The values obtained from ITC and fluorimetric data for *K*_b,1_ are consistent (note significant uncertainty in *K*_b,1_) and are of the same order of magnitude. For *K*_b,2_, the constant resulting from the fit of the fluorimetry data is an order of magnitude smaller than the binding constant corresponding to the ITC data. However, such differences are common when addressing macromolecular binding^65^ and in our case do not influence the conclusions drawn from Δ*G*_b,2_^⊖^. Both ITC and fluorimetry give the same trend in binding strength, that is, *K*_b,2_ < *K*_b,1_.

### Influence of the BSA–NaPSS Mixing Order on the Thermodynamics of Complexation

The results discussed so far apply to NaPSS-to-BSA titration. However, it has been documented that protein–PE complexation may depend on the order in which the solutions of the protein and PE are mixed.^13^ Therefore, we also performed the reverse titration experiment in which a NaPSS solution was titrated with the BSA solution (a BSA-to-NaPSS titration). Both BSA and NaPSS were dissolved in the same phosphate buffer as that used in the NaPSS-to-BSA titration (*I*_total_ = 20 mM, pH = 8.0). The comparison between the binding isotherms of the NaPSS-to-BSA and BSA-to-NaPSS titrations is shown in Figure S4 in the Supporting Information file. Similar to that in NaPSS-to-BSA titration, complexation in case of BSA-to-NaPSS titration is also exothermic and characterized by two binding processes. In BSA-to-NaPSS titration, the divide between the structuring regimes is somewhat less ambiguous than that in the NaPSS-to-BSA case. The thermodynamic parameters obtained from the TSIS model fit of the BSA-to-NaPSS binding isotherm are summarized in Table 1. To distinguish between the parameters of the NaPSS-to-BSA titration and those of the BSA-to-NaPSS titration, the latter are in the text decorated with apostrophe (e.g., *K*_b,*i*_ denotes the binding constant of the NaPSS-to-BSA titration, while *K*_b,*i*_^′^ stands for the BSA-to-NaPSS titration). From the differences in numbers given in Table 1, we see that the thermodynamic parameters of the first and second processes depend on the mixing order. The binding constant of the first binding process is up to 2 times larger than the *K*_b,1_ while it is about 16 times smaller for the second binding process . SANS measurements reported by Chodankar et al.^61^ showed similar asymmetry with respect to the order of mixing in the BSA/NaPSS system, but the results are not directly comparable due to different experimental conditions (they performed experiments at *T* = 30 °C, and MW of NaPSS was 100 kDa, pH = 6.5, *I* = 500 mM). Since the separation of the two binding processes is slightly better in the BSA-to-NaPSS titration and *K*_b,1_^′^ > *K*_b,1_, we can assume that the formation of intrapolymer complexes is on average more extensive at lower molar ratios (low BSA concentration) than that in NaPSS-to-BSA titration (high BSA concentration). This is plausible because BSA has two positive binding domains (Figure S2—left) where association with NaPSS can occur at pH = 8.0. Naturally, binding to both domains should be more pronounced with an excess of NaPSS, resulting in stronger binding. The same is not true for NaPSS-to-BSA titration, as complexation occurs in excess of BSA, where binding would favor the binding site that allows stronger association (either A or B; Figure S2). As with the NaPSS-to-BSA titration, the lack of a well-defined plateau on the reverse titration binding isotherm prevented unambiguous determination of the binding stoichiometry and the standard binding enthalpy change of the first binding process (*n*_1_^′^, ).

The second binding process is connected with the association of intrapolymer complexes and proceeds by the addition of the titrant (either BSA or NaPSS). Since NaPSS molecules are larger and more extended, they act as better cross-linkers compared to the smaller BSA molecules. This could be the reason why the second binding process in NaPSS-to-BSA titration has about a 16 times higher binding constant than that in the reverse titration . In addition, cross-linking NaPSS molecules provide more potential binding sites for BSA molecules, which are in excess in NaPSS-to-BSA titration. Consequently, more BSA molecules are complexed in the second binding process (7–10 proteins per PE chain) than in the BSA-to-NaPSS titration, where the binding stoichiometry is *n*_2_^′^ = 3.68 ± 0.06 (3–4 proteins per PE chain). The binding enthalpy change for the second binding process, Δ*H*_b,2_^⊖^′ = (−43.7 ± 0.6) kJ/mol, is about 9 times lower than the Δ*H*_b,2_^⊖^. This means that less favorable charge–charge interactions occur when BSA molecules act as cross-linkers. The entropic contribution for the second binding process is also less unfavorable (*T*Δ*S*_b,2_^⊖^′ = (−8.8 ± 0.7) kJ/mol, about 45 times lower than *T*Δ*S*_b,2_^⊖^), which is probably the consequence of a smaller penalty upon association of intrapolymer complexes through BSA molecules compared to that upon the association by NaPSS molecules, which lose more configurational entropy during the second binding process of NaPSS-to-BSA titration.

### Conformational Changes of BSA and Binding Site Determination

**Figure 3:**
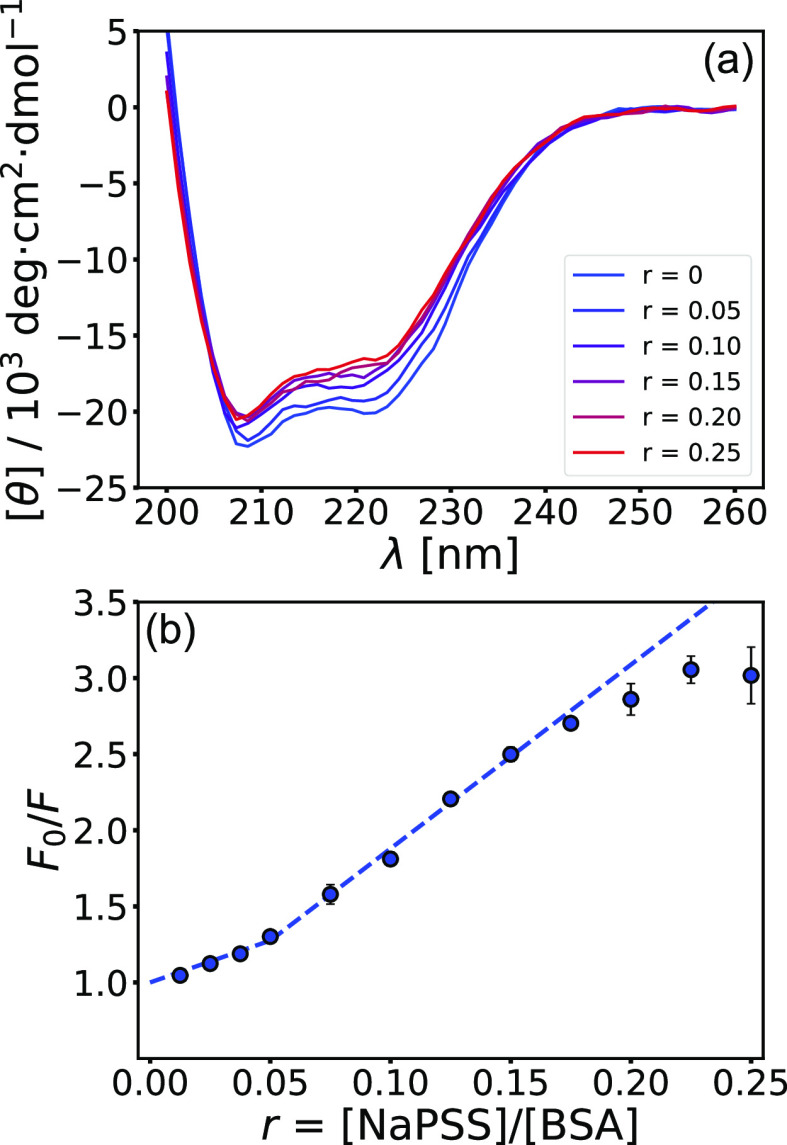
The formation of BSA–NaPSS complexes leads to conformational changes of the protein. (a) CD spectra of 2.5 μM BSA at different NaPSS/BSA molar ratios, *r*, and (b) Stern–Volmer plot for quenching of tryptophan fluorescence by NaPSS (λ_ex_ = 295 nm, *c*_BSA_ = 2 μM) recorded at 350 nm. Dashed lines are linear fits. All solutions were prepared in phosphate buffer (*I*_total_ = 20 mM, pH = 8.0), and data were collected at 25 °C.

To investigate whether the protein undergoes any conformational changes upon complexation with NaPSS, the CD spectra of BSA were recorded at different NaPSS/BSA molar ratios, *r*. As shown in Figure 3a, some changes in the secondary structure of BSA occur upon complexation. BeStSel software^54^ was used to analyze the spectra. The estimated secondary structure content as a function of *r* is given in Table S2 in the Supporting Information file. A decrease in the amount of the α-helical content of BSA and the appearance of β-sheets with increasing *r* can be seen. Conformational changes occur at molar ratios corresponding to both binding processes, but the decrease in the α-helical content is more pronounced at *r* ≳ 0.05. Since there are two positive charge patches on the BSA surface, BSA–NaPSS binding may occur at different parts of the BSA surface (binding sites). This could be a distinguishing feature between the two binding processes.

The formation of BSA–NaPSS complexes is accompanied by changes of the molecular structure of BSA, which can be monitored by UV–vis spectroscopy. As can be seen in Figure S5a, complex formation leads to an increase in the solution absorbance, which is accompanied by a shift of the absorption maximum toward shorter wavelengths with increasing *r*. However, these changes are related to the increasing concentration of NaPSS, which becomes evident upon subtracting the spectra of corresponding NaPSS-buffer solutions (Figure S5b). Minute changes of the absorbance at 280 nm (Figure S5b; inset) could be related to changes of the absorptive properties of BSA–NaPSS complexes reflected in the above-mentioned conformational changes. However, these changes are too small to provide any additional information in terms of binding region localization.

Fluorescence emission spectroscopy offers a viable tool for evaluating conformational changes around specific fluorophores. By exciting only tryptophan residues (λ_ex_ = 295 nm), changes in their emission spectra can be correlated with changes in their molecular environment. BSA has two tryptophan residues: Trp-134, which is located near the binding domain A and more close to the protein surface, and Trp-213, which is more buried and located within the binding domain A (see Figure S2). Binding domain B is not located near either Trp residue (of the two Trp residues, domain B is closer to Trp-213). Assuming that the binding of NaPSS mainly alters the conformation of BSA in the vicinity of the binding site (charge patch), this could be reflected in changes in the molecular environment of the tryptophans. Figure S6 (*T* = 25 °C) in the Supporting Information file shows the emission spectra of BSA at different *r* values, while the normalized fluorescence intensity at 350 nm as a function of the molar ratio is shown in Figure 3b (Stern–Volmer plot). In Figure 3b, three different regimes are observed: The first regime occurs at 0 < *r* ≲ 0.05, which is the approximate range where the first binding process is thought to occur (see Figure 2). The second regime ranges from *r* ≈ 0.05 to approximately 0.15, which can be associated with the second binding process. The deviation from linearity at higher quencher concentrations (third regime) is often observed for proteins with two different fluorophore populations. In our case, this occurs at *r* ≳ 0.15 and is related to the saturation of the protein binding sites as a constant signal is reached caused by the emission of the more buried Trp-213 residue. In contrast to the calorimetric data, the separation between the two binding processes is more clearly expressed in the Stern–Volmer plot (note the change in the slope at *r* ≈ 0.05). The Stern–Volmer quenching constants are equal to *K*_SV,1_ = (3.2 ± 0.2) × 10^3^ and *K*_SV,1_ = (12.1 ± 0.6) × 10^3^ L/mol for the first and second binding processes, respectively. A clear separation between the two regimes and differences in the quenching constants indicate that changes in the molecular environment of the Trp residues correlate with changes in the secondary structure of BSA and the two binding processes detected in the ITC titration.

Fluorescence quenching can be classified as static, dynamic (collisional), or a combination of both processes. In the case of dynamic quenching, the quencher (NaPSS) must diffuse to the fluorophore (tryptophan) during the lifetime of the excited state, leading to a non-radiative relaxation through collisions. Static quenching, on the other hand, results in the formation of non-fluorescent ground-state complexes. The bimolecular quenching rate constant can be calculated (*k*_q_ = *K*_SV_/τ_0_; see eq 6) and compared to the value of a typical diffusion-controlled interaction to estimate the predominance of either mechanism. For the diffusion-controlled quenching process with a protein, the maximum value of *k*_q_ is approximately 2 × 10^10^ L/mol s.^51^ Rate constants in our case are *k*_q,1_ = 5.5 × 10^11^ L/mol s and *k*_q,1_ = 2.1 × 10^12^ L/mol s for the first and second binding process, respectively. These values are too large to indicate significant quenching due to collisions alone, indicating the formation of ground-state complexes. Static and dynamic quenchings can also be distinguished by their differing dependence on temperature.^51^ Since complex formation between BSA and NaPSS is driven by electrostatic forces (see explanation later on), fluorescence quenching is expected to decrease at higher temperatures due to weaker interactions between the macromolecules. On the other hand, if the predominant quenching mechanism is dynamic, quenching should increase at higher temperatures due to an increase in collisions between the quencher and fluorophore. The fluorescence emission spectra as a function of *r* at 15, 25, and 35 °C are shown in Figure S6, and the corresponding Stern–Volmer plots are shown in Figure S7. The Stern–Volmer quenching constants for different temperatures are given in Table S3. The quenching constants for both binding processes (*K*_SV,1_, *K*_SV,2_) decrease as temperature is increased from 15 to 35 °C, which confirms the static quenching mechanism as the dominant origin for the fluorescence quenching.

Considering the analyzed experimental data (ITC, absorbance and emission spectra, CD measurements) and charge distribution on the protein surface (Figure S2—left), we can speculate that the first binding process (i.e., formation of intrapolymer complexes) is associated with NaPSS binding predominantly to the more accessible binding patch/domain B. This is accompanied by minor conformational changes that likely affect the molecular environment of Trp-213 (*K*_SV,1_), which is located closer to domain B. The second binding process (i.e., formation of interpolymer complexes) could be facilitated by the binding of NaPSS to the more buried positive patch/domain A, which is located closer to both Trp residues. More efficient binding of NaPSS to the less accessible domain A requires larger conformational changes, which manifest themselves in larger changes in the molecular environment of probably both Trp residues (*K*_SV,2_) located in the vicinity of this domain.

The presence of two positively charged domains on the BSA surface at pH = 8.0 (*I*_total_ = 20 mM) and a strongly exothermic effect upon BSA–NaPSS mixing (Δ*H* < 0) suggest that electrostatic interactions are the main driving force of complexation on the “wrong side”. Upon comparing the complexation in the BSA/NaPSS system with the complexation between BSA and other, more hydrophilic PEs containing −SO_3_^–^ groups [e.g., PAMPS,^17,29^ poly(vinyl sulphate),^17^ PAGE–SO_3_Na,^33^] we can also conclude that complexation above the isoionic point of BSA can be explained without invoking the hydrophobic character of NaPSS. In our work, atactic NaPSS was used, where the random orientation of the functional groups with respect to the PE backbone further reduces the probability of non-electrostatic binding. In addition, the surface of BSA does not have major hydrophobic domains that could favor significant short-range non-electrostatic interactions between the lipophilic regions of the protein and the apolar backbone of NaPSS (see the hydrophobicity map of BSA displayed in Figure S2—right). By increasing the ionic strength of the solution, however, the electrostatic interactions become screened; therefore, short-ranged interactions could potentially be more significant at higher ionic strength. In the next section, we examine the BSA/NaPSS complexation as a function of total ionic strength of the solution and the influence of low-molecular weight salts (NaCl, NaBr, and NaI) on the interactions between the protein and PE at pH = 8.0.

In this study, fatty acid-free BSA was used. However, it is known that BSA functions as the major fatty acid-binding protein in blood plasma.^66^ The question arises whether the conclusions of this study can differ if BSA samples with bound fatty acids are used. Complex formation between BSA and NaPSS is electrostatically driven, so fatty acids located in the hydrophobic pockets of BSA would have no effect on the binding between BSA and NaPSS. The positive patches on the BSA surface which facilitate binding between BSA and NaPSS under the conditions studied are quite large (domains A and B in Figure S2) and would not be screened by the carboxylate groups of the fatty acids. Since a NaPSS molecule has multiple neighboring −SO_3_^–^ groups, it would likely displace the bound fatty acid due to the stronger electrostatic attraction. We believe that the presence of fatty acids would therefore not affect our conclusions. A more detailed commentary on this topic can be found in the Supporting Information file (Figure S8).

### Influence of Salts on the BSA–NaPSS Complexation—The Hofmeister Effect

Due to the electrostatic nature of the BSA–NaPSS interaction, the ionic strength of the medium can have a strong influence on the complexation. We performed NaPSS-to-BSA titrations with solutions of different ionic strengths, *I*_total_. Sodium chloride was added to the phosphate buffer (*I*_buffer_ = 20 mM) to adjust the total ionic strength of the medium, which is equal to the sum of the ionic strength of the buffer and the added salt, *I*_total_ = *I*_buffer_ + *I*_salt_. pH of all solutions was 8.0 at 25 °C.

**Figure 4:**
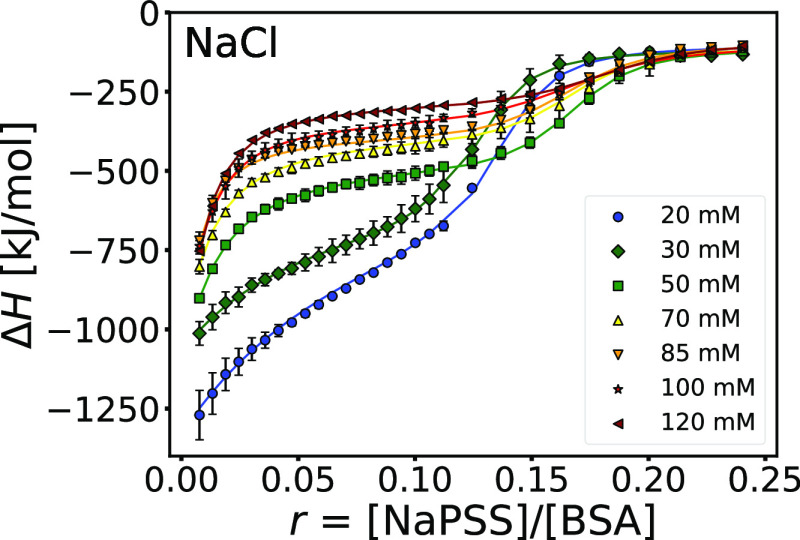
The presence of NaCl has a different impact on the two binding processes in BSA–NaPSS complexation on the “wrong side”. The binding isotherms for the NaPSS-to-BSA titration at various total ionic strengths. The fit of a TSIS binding model is shown as solid lines. All solutions were prepared in phosphate buffer (*I*_buffer_ = 20 mM, pH = 8.0), and total ionic strength was regulated by addition of NaCl, *I*_total_ = *I*_buffer_ + *I*_NaCl_. Data were collected at 25 °C.

Figure 4 shows ITC binding isotherms for a NaPSS-to-BSA titration at various *I*_total_ (from 20 to 120 mM). The heat release associated with BSA–NaPSS complexation decreases with increasing ionic strength (mixing is less exothermic). This is the result of electrostatic screening caused by the presence of salt ions (NaCl), which leads to less energetically favorable electrostatic interactions between BSA and NaPSS. Interestingly, the separation between the two binding processes becomes clearer with increasing *I*_total_, indicating that the two binding processes respond differently to electrostatic screening. This could be due either to a different modulation of the electrostatic interactions of the two processes or to the presence of short-range non-electrostatic interactions which become more prevalent at higher ionic strengths.

**Figure 5:**
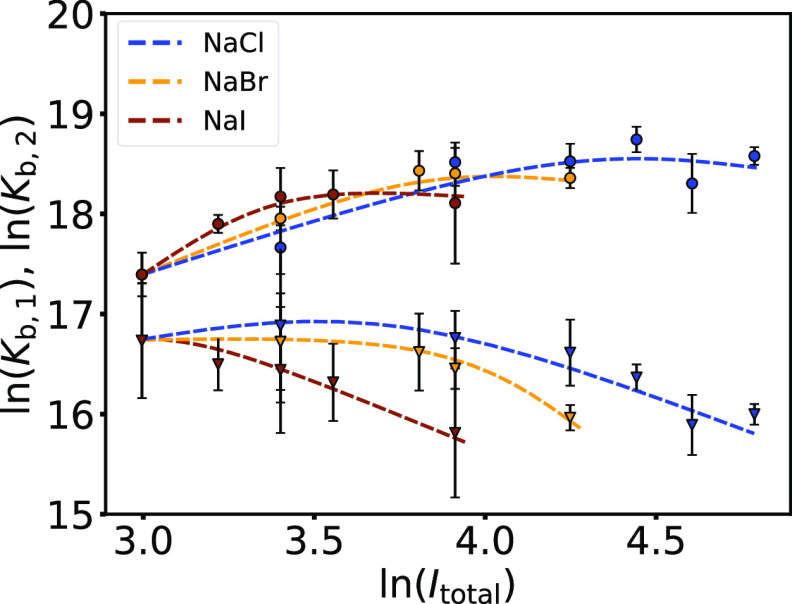
The effect of salts on the complexation between BSA and NaPSS is nonmonotonic*.* Dependence of *K*_b,1_ (circles) and *K*_b,2_ (triangles) on the total ionic strength, *I*_total_. Different sodium salts—NaCl (blue), NaBr (yellow), and NaI (brown)—were used to adjust the ionic strength of the solution (20 mM phosphate buffer, pH = 8.0; *cf*. Tables 2, S5, and S6). Data were collected at *T* = 25 °C. Lines are guides to the eye.

The thermodynamic parameters of the TSIS model, obtained by fitting the binding isotherms shown in Figure 4, are summarized in Table 2. The trends of the dependence of the binding constants of the first and second binding processes (*K*_b,1_ and *K*_b,2_, respectively) on the total ionic strength are also shown in Figure 5 (blue symbols and line apply to the NaCl case). The *K*_b,1_ shows a non-monotonic behavior: the binding constant initially increases with increasing *I*_total_ and reaches a maximum value. A similar trend holds for *K*_b,2_, although the maximum is shifted to a much lower *I*_total_ and is not well pronounced in the case of solutions with added NaCl. With further increase in *I*_total_, the values of the binding constant decrease. Such dependencies for protein–PE complexation have been described previously (see refs (5), (21), (22), and (42)) and usually show a maximum at a certain interval of ionic strengths (5–30 mM). In our case, the dependence for the first binding process seems to have a maximum at *I*_total_ ≈ 70–85 mM for solutions containing NaCl. The dependence for the second binding process is similar but with a maximum at lower ionic strengths (*I*_total_ ≈ 20–50 mM). Due to the limited number of data points and rather large uncertainties, the exact location of the maximum is difficult to determine.

**Table 2:** Thermodynamic Parameters of the First and Second Binding Processes Obtained by Fitting the TSIS Model to the Binding Isotherm of the NaPSS-To-BSA Titration as a Function of Total Ionic Strength (*cf*. Table 1 for Description of Parameters)^a^

NaCl	I. binding process		II. binding process				
*I* [mM]	*K*_b,1_ × 10^–7^	Δ*G*_b,1_^⊖^ [kJ/mol]	*K*_b,2_ × 10^–7^	Δ*G*_b,2_^⊖^ [kJ/mol]	*n*_2_	Δ*H*_b,2_^⊖^ × 10^–2^ [kJ/mol]	*T*Δ*S*_b,2_^⊖^ ×10^–2^ [kJ/mol]
20	4 ± 1	–43.4 ± 0.6	2 ± 1	–42 ± 1	0.12 ± 0.02	–4 ± 2	–4 ± 2
30	5 ± 1	–43.8 ± 0.7	2 ± 2	–42 ± 2	0.11 ± 0.02	–5 ± 2	–4 ± 2
50	11 ± 2	–45.9 ± 0.4	1.9 ± 0.5	–41.5 ± 0.7	0.145 ± 0.007	–3.8 ± 0.2	–3.4 ± 0.2
70	11 ± 2	–45.9 ± 0.4	1.6 ± 0.5	–41.2 ± 0.8	0.144 ± 0.009	–2.9 ± 0.2	–2.5 ± 0.2
85	14 ± 2	–46.5 ± 0.3	1.3 ± 0.2	–40.6 ± 0.3	0.143 ± 0.004	–3.07 ± 0.06	–2.67 ± 0.06
100	9 ± 3	–45.4 ± 0.7	0.8 ± 0.2	–39.4 ± 0.7	0.141 ± 0.007	–2.5 ± 0.2	–2.1 ± 0.2
120	12 ± 1	–46.0 ± 0.2	0.89 ± 0.09	–39.7 ± 0.3	0.154 ± 0.002	–2.15 ± 0.04	–1.75 ± 0.04

a All solutions were prepared in phosphate buffer (*I*_buffer_ = 20 mM, pH = 8.0), and total ionic strength was adjusted by addition of NaCl. Data were collected at *T* = 25 °C.

BSA has a net negative charge at pH = 8.0. However, because of the heterogeneous charge distribution on its surface (two positively charged domains shown in Figure S2—left), complex formation between BSA and NaPSS can be facilitated by the interaction between positive domains of the protein and the negatively charged −SO_3_^–^ groups of NaPSS. This favorable electrostatic interaction can overcome the overall repulsion between the two negatively charged macromolecules.^19^ The result is a coexistence of short-range attractive and long-range repulsive electrostatic forces (SALR) between the two macromolecules, which can be regulated by ionic strength at a constant pH. A relatively simple and qualitative model, first applied to the complexation of gelatin with NaPSS,^67^ was later extended to the complexation between proteins and PEs: BLG with NaPSS, PAMPS^21^, and poly(vinyl sulphate)^68^ as well as BSA–heparin and insulin–heparin.^22^ The model takes into account the interactive potential (see ref (21)) between the negatively charged PE segment near the positive domain of the protein surrounded by negatively charged domains. In the model, the interaction energy changes solely as a function of the Debye screening length , leading to a maximum value in the dependence of *K*_b_ on *I*_total_.

**Figure 6:**
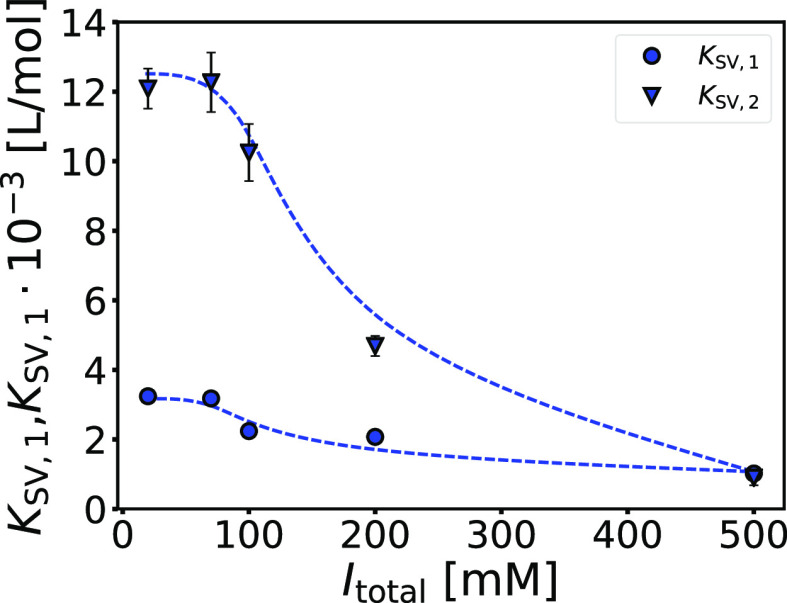
The effect of NaCl on the Stern–Volmer quenching constants is nonmonotonic for both binding processes*.* Stern–Volmer quenching constants for first, *K*_SV,1_, and second binding processes, *K*_SV,2_, as a function of total ionic strength, *I*_total_. Solutions were prepared in phosphate buffer (pH = 8.0) with different ionic strengths regulated with NaCl, λ_ex_ = 295 nm, *T* = 25 °C.

As discussed in the section BSA–NaPSS Complexation without the Co-Solute Present, BSA–NaPSS complexation at low ionic strengths (*I*_total_ = 20 mM) is driven solely by electrostatic forces. However, it is known that the strength of short-range non-electrostatic interactions increases with increasing ionic strength as the Coulomb interactions are increasingly screened. A non-monotonic behavior of the two binding constants as a function of *I*_total_ (Figure 5) could also be indicative of a balance between electrostatic and non-electrostatic interactions. The nature of the interactions depends strongly on the protein–PE system under study.^17,21,29,30,33^ For example, the interaction between BLG and NaPSS is the result of electrostatic interactions only.^21,29,30,69^ However, the surface of BSA has more non-polar regions than that of BLG, which could become important in the complexation between BSA and NaPSS at higher ionic strengths. As can be seen from the hydrophobicity map of BSA shown in Figure S2 (right), scattered hydrophobic regions could still provide potential binding sites for non-polar molecules. As described in the literature, adsorption of BSA onto hydrophobic surfaces is accompanied by conformational changes,^70,71^ and it is not unreasonable to assume that if short-range non-electrostatic forces are present for the BSA–NaPSS system at higher ionic strengths, this could be detected by looking at conformational changes. The CD spectra of BSA/NaPSS solutions at different *r* and different total ionic strengths (regulated by NaCl) are shown in Figure S9 in the Supporting Information file, while the estimated alpha-helical content of BSA is given in Table S4. The extent of conformational changes decreases with increasing *I*_total_ and is no longer detected at *I*_total_ = 500 mM. Since no pronounced conformational changes with respect to *r* occur at larger ionic strengths where electrostatic interactions are sufficiently screened, we can conclude that conformational changes of BSA are solely a consequence of electrostatic interactions. As discussed in the subsection Conformational Changes of BSA and Binding Site Determination, conformational changes of BSA are reflected in changes in the molecular environment of its tryptophan residues, which will also depend on *I*_total_. The fluorescence emission spectra as a function of *r* at different *I*_total_ are shown in Figure S10, and the corresponding Stern–Volmer plots are shown in Figure S11. The Stern–Volmer quenching constants (*K*_SV,1_ and *K*_SV,2_) for different *I*_total_ are given in Table 3 and plotted in Figure 6. A similar non-monotonic dependence as in the case of the binding constants shown in Figure 5 can be seen. Changes in the molecular environment of tryptophans for the second binding process are more sensitive to changes in *I*_total_, suggesting that this process is indeed related to the binding of NaPSS to the more buried binding domain A (requiring larger conformational changes), whereas the opposite is true for the first binding process, where NaPSS presumably binds predominantly to domain B (requiring smaller conformational changes). Moreover, the electrostatic potential maps at different ionic strengths shown in Figure S12 in the Supporting Information file clearly indicate the presence of both positive-charge domains (A and B) even at higher ionic strengths (*I*_total_ = 500 mM), confirming the dominance of electrostatic interactions in the BSA/NaPSS system.

**Table 3:** Stern–Volmer Quenching Constants for the First and Second Binding Process (*K*_SV,1_ and *K*_SV,2_, Respectively) Obtained from the Stern–Volmer Plot (2.0 μM BSA; cf. Figure S11)^a^

*I* [mM]	*K*_SV,1_ × 10^–3^ [L/mol]	*K*_SV,2_ × 10^–3^ [L/mol]
20	3.2 ± 0.2	12.1 ± 0.6
70	3.2 ± 0.1	12.3 ± 0.9
100	2.2 ± 0.2	10.2 ± 0.8
200	2.07 ± 0.07	4.7 ± 0.3
500	1.02 ± 0.05	0.9 ± 0.2

a All solutions were prepared in the phosphate buffer (pH = 8.0) with different ionic strengths regulated with NaCl, λ_ex_ = 295 nm, *T* = 25 °C.

A balance between SALR repulsive electrostatic forces is responsible for the nonmonotonicity in the dependence of *K*_b,*i*_ on *I*_total_ for both binding processes (Figure 5). Consequently, an optimal binding strength between BSA and NaPSS exists at *I*_total_ ≈ 70–85 and 20–50 mM for the first and second binding process, respectively, in the case of solutions with pH = 8.0 and containing NaCl. The location of the maximum value of the binding constant (strongest protein–PE interactions) depends also on the persistence length (chain stiffness) of the polyanion. Polyanions with large charge density are rigid molecules. Consequently, the repulsive regime will be more far-reaching and will extend to higher ionic strengths (Figure 5). In other words, a more flexible polyanion conforms more readily to a bound configuration of lower energy and thus has a larger binding constant when the charges on the PE are more screened (at higher ionic strengths, see refs (72) and (73)). Changes of the PE’s chain stiffness are also caused by the complexation with proteins, which explains why the maximum of the second binding process is located closer to ionic strengths reported in the literature (5–30 mM^22^). At this stage, BSA molecules are already bound to NaPSS (formation of intrapolymer complexes).

So far, we have discussed the influence of the ionic strength of the solution on the complexation of BSA and NaPSS at pH = 8.0. The ionic strength of the buffered solution was adjusted with sodium chloride. Since the ions of the low-molecular weight salts also compete with the charged sites on the macromolecules, this could lead to salt-specific effects on the protein–PE complexation. To investigate the role of the chemical identity of the salt anions in BSA–NaPSS complexation, sodium bromide and sodium iodide were also used to regulate total ionic strengths. The binding isotherms for solutions with NaBr and NaI are shown in Figure S13 in the Supporting Information file. The thermodynamic parameters of the TSIS model are given in Tables S5 and S6 for NaBr and NaI, respectively. In addition to the case of NaCl discussed above, the dependencies of the binding constants of the first and second processes on *I*_total_ for BSA/NaPSS systems containing NaBr and NaI are shown in Figure 5. As can be seen from Figure 5, the nonmonotonicity in *K*_b,*i*_ versus *I*_total_ for NaBr and NaI as co-solutes is similar to that for NaCl, but the maxima are shifted to lower ionic strengths. This effect can be explained in light of the chaotropic character of the salt anion, which decreases in the order I^–^ > Br^–^ > Cl^–^. It is known that larger and more polarizable anions have a tendency toward positively charged regions of proteins.^74,75^ Therefore, a lower concentration of the salt with the more chaotropic anion (I^–^, Br^–^) is required to achieve the same screening effect as that achieved with the salt with the less chaotropic anion (Cl^–^). Consequently, the coexistence between the SALR repulsive electrostatic regimes shifts to lower ionic strengths (the shift does not imply non-specific interaction between BSA and NaPSS). Therefore, the maximum for the first binding process can be identified at *I*_total_ ≈ 50 mM and *I*_total_ ≈ 30 mM for NaBr and NaI, respectively. The dependence for the second binding process also resembles that of NaCl, but the maximum probably shifts to *I*_total_ < 20 mM, which was outside the range of the experiments performed in this work.

**Figure 7:**
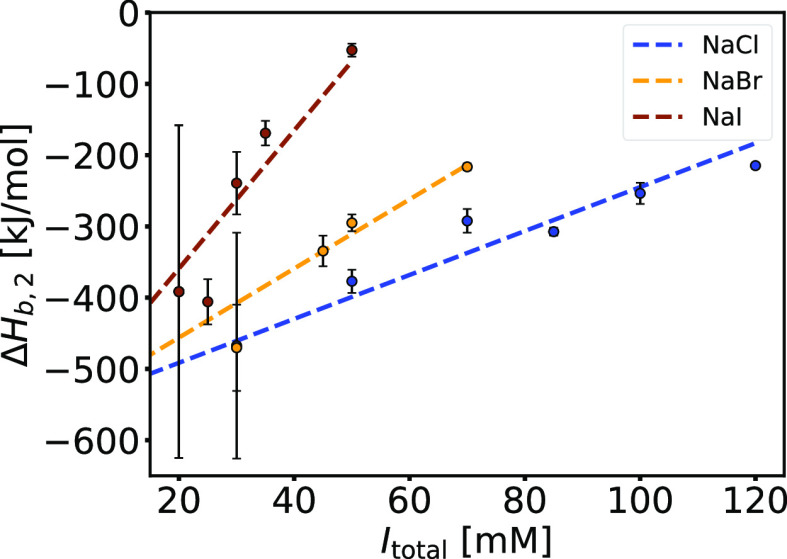
Enthalpic changes associated with the formation of interpolymer BSA/NaPSS complexes decrease with ionic strength and with the chaotropic character of the salt anion. Dependence of the standard binding enthalpy change of the second binding process, Δ*H*_b,2_^⊖^, on the total ionic strength, *I*_total_, at *T* = 25 °C, obtained by the TSIS binding model (*cf*. Tables 2, S5, and S6). The ionic strength of the phosphate buffer (pH = 8.0) was adjusted by adding NaCl (blue), NaBr (yellow), or NaI (brown).

From a thermodynamic point of view, the overall heat release associated with the formation of intra- and interpolymer complexes decreases with increasing *I*_total_ and with the chaotropic character of the anion (see Figures 4 and S13). The dependence of the standard binding enthalpy change for the second binding process, Δ*H*_b,2_^⊖^, as a function of total ionic strength (controlled with NaCl, NaBr, or NaI) is shown in Figure 7. For a given salt system, Δ*H*_b,2_^⊖^ decreases (becomes less exothermic) with increasing *I*_total_. For a given ionic strength, Δ*H*_b,2_^⊖^ is most exothermic for systems with NaCl and least exothermic for systems with NaI. In systems with more chaotropic salt anions, the extent of favorable charge–charge interactions is smaller. The changes in the standard entropy of binding of the second process, *T*Δ*S*_b,2_^⊖^, upon increasing *I*_total_ are shown for systems with NaCl, NaBr, and NaI in Figure S14 in the Supporting Information file. The unfavorable entropic contribution of the second binding process shows similar trends as Δ*H*_b,2_^⊖^: the less NaPSS is bound to BSA, the less configurational freedom of PE is lost (entropically more favorable).

**Figure 8:**
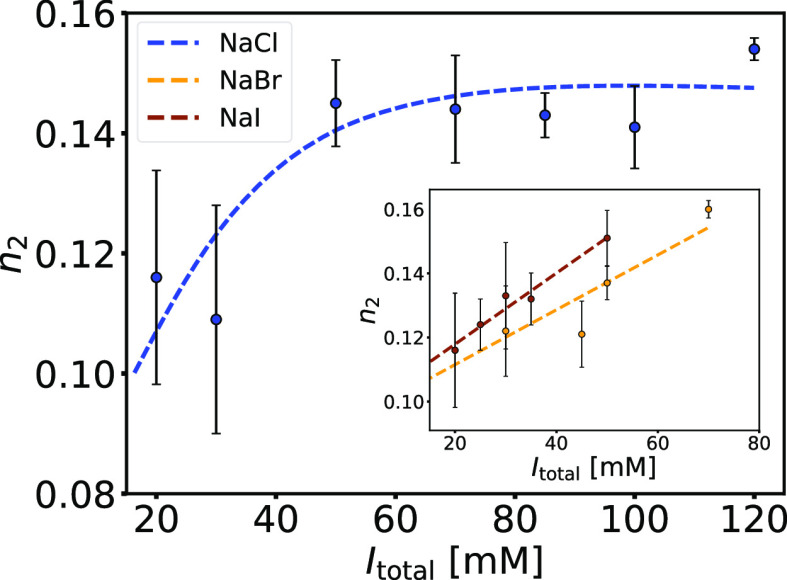
The number of BSA molecules bound to NaPSS decreases with *I*_total_ and with the chaotropic character of the salt anion. Dependence of the binding stoichiometry of the second binding process, *n*_2_, on the total ionic strength, *I*_total_, at *T* = 25 °C, obtained by the TSIS binding model (*cf*. Tables 2, S5, and S6). The ionic strength of the phosphate buffer (pH = 8.0) was adjusted by adding NaCl (blue), NaBr (yellow; inset), or NaI (brown; inset).

The dependence of the binding stoichiometry of the second binding process, *n*_2_, on *I*_total_ is shown in Figure 8. *n*_2_ increases with increasing *I*_total_ (note that 1/*n*_2_ represents the average number of BSA molecules bound to one NaPSS chain). For solutions containing NaBr and NaI, the increase is linear for all ionic strengths studied. For solutions containing NaCl, *n*_2_ reaches a constant value at larger ionic strengths. We speculate that the modulating effect of chloride ions on binding affinity is mainly due to electrostatic screening and does not directly affect the binding domains on the protein. Consequently, *n*_2_ depends on the balance between SALR repulsive electrostatic forces at the ionic strengths studied. For *I*_total_ ≲ 40 mM, both coexist, and screening by chloride ions manifests itself in an increase in *n*_2_ upon salt addition, but above approximately 50 mM, attractive interactions gain prevalence, and addition of salt does not affect *n*_2_. Since these interactions are very strong, we can assume that higher ionic strengths are required to modulate the binding stoichiometry determined by ITC experiments. The same is not true for the modulating effect of NaBr and NaI since chaotropic anions have an affinity for the positive binding domains of BSA—they essentially act as competitors to the sulfonic groups of NaPSS. Since experiments with NaBr and NaI at higher ionic strengths were not possible (the second binding process starts disappearing—see Figure S13), we can only assume that similar deviations from linearity as those with NaCl would also be observed with NaBr or NaI at higher ionic strengths (*I*_total_ ≳ 70 mM).

### Influence of Sugars on the BSA–NaPSS Complexation

In protein solutions, sugars are quite known for their bioprotective role and usually reduce the influence of external stressors (changes in temperature, chemical denaturants) on either the protein’s solution or conformational stability. The mechanism usually responsible for the stabilizing role is the so-called preferential hydration mechanism, where sugars are excluded from the protein surface, thus enforcing a stronger hydration of the protein. This mechanism was shown to hold true for sucrose but not for its structural analogue, sucralose, which tends to interact directly with the protein surface (preferential interaction mechanism).^32,76^

The chemical modification (chlorination) of sucrose (see Figure 1b,c) results in different physico-chemical properties and different water-structuring abilities. By using a combination of molecular dynamics and fluorescence measurements, we concluded that sucralose molecules form a coating around the protein surface, a property which manifests itself in regard to protein–protein (aggregation) and protein–PE (complexation) interactions.^32^ In addition, this effect depends on the heterogeneity of the protein surface (e.g., differently for lysozyme^76^ or BSA^32^) and the pH of the medium.

**Figure 9:**
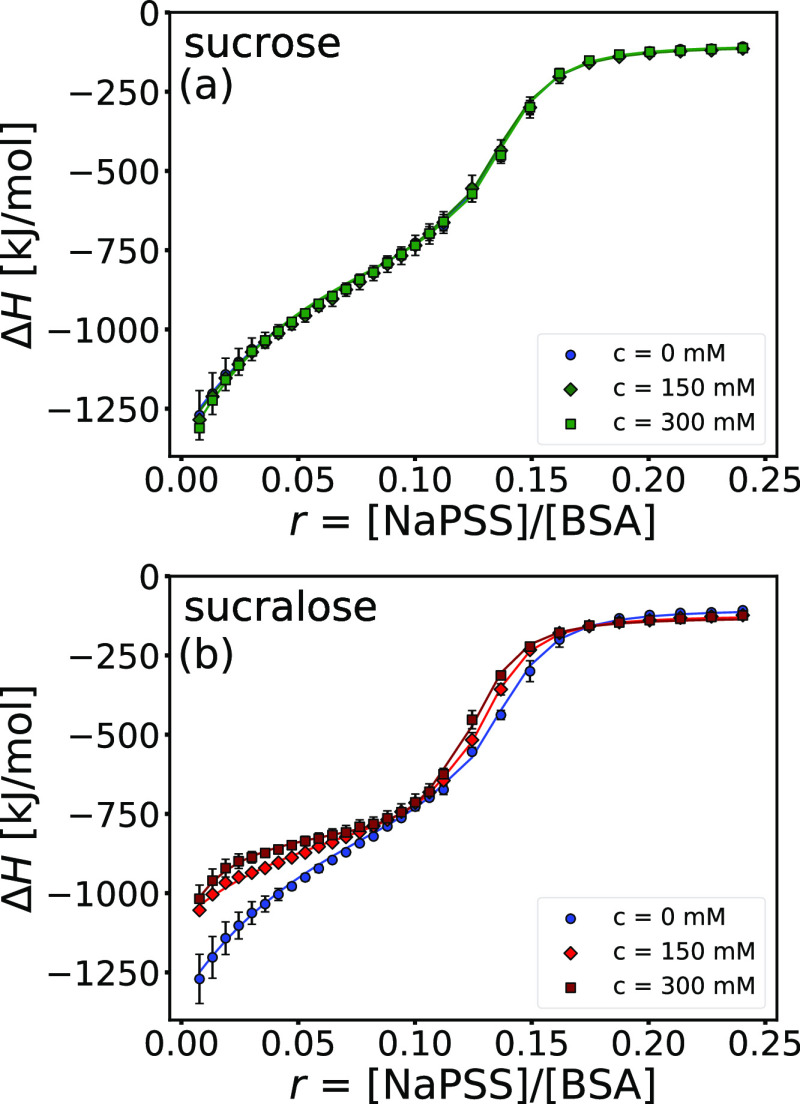
The presence of sucrose does not affect the BSA–NaPSS complexation on the “wrong side”, whereas sucralose slightly promotes the complexation at this pH. The binding isotherms for the NaPSS-to-BSA titration at various (a) sucrose and (b) sucralose concentrations (0, 150, and 300 mM). The fit of a TSIS binding model is shown as solid lines. All solutions were prepared in phosphate buffer (*I*_total_ = 20 mM, pH = 8.0). Data were collected at 25 °C.

We performed the NaPSS-to-BSA ITC experiments with solutions (20 mM phosphate buffer with pH = 8.0) that also contained various concentrations of sucrose or sucralose. The binding isotherms for BSA–NaPSS systems containing 150 and 300 mM sucrose and sucralose are shown in Figure 9 (the binding isotherm of the sugar-free system is also shown for comparison). The thermodynamic parameters of the TSIS model fitted to the binding isotherms are given in Tables 4 and 5 for sucrose and sucralose, respectively. From Figure 9a and the thermodynamic parameters in Table 4, it is clear that the presence of sucrose has no effect on BSA–NaPSS complexation. This is not surprising since sucrose molecules are preferentially excluded from the BSA surface,^32^ and a stronger hydration of the protein is not sufficient to affect the electrostatic forces between BSA and NaPSS. The same is not true for sucralose (Figure 9b and Table 5), as it interacts directly with the BSA surface.^32^ The binding constant associated with the first binding process, *K*_b,1_, actually increases in the presence of sucralose, indicating stronger binding between BSA and NaPSS in the presence of sucralose. The increase in the binding constant of the second process, *K*_b,2_, is less pronounced, although no definite conclusion can be drawn because of the large uncertainties. A stronger interaction between BSA and NaPSS in the presence of sucralose seems to contradict the established role of sucralose as a stabilizing agent when it comes to phase separation.^32^ However, we note that the propensity of sucralose molecules depends strongly on the pH of the medium and is different for pH > *pI*_BSA_ and pH ≈ *pI*_BSA_.

**Table 4:** Thermodynamic Parameters of the First and Second Binding Processes Obtained by Fitting the TSIS Model to the Binding Isotherm of the NaPSS-To-BSA Titration as a Function of the Sucrose Concentration (*cf*. Table 1 for Description of Parameters)^a^

sucrose	I. binding process		II. binding process				
*c* [mM]	*K*_b,1_ × 10^–7^	Δ*G*_b,1_^⊖^ [kJ/mol]	*K*_b,2_ × 10^–7^	Δ*G*_b,2_^⊖^ [kJ/mol]	*n*_2_	Δ*H*_b,2_^⊖^ × 10^–2^ [kJ/mol]	*T*Δ*S*_b,2_^⊖^ ×·10^–2^ [kJ/mol]
0	4 ± 1	–43.4 ± 0.6	2 ± 1	–42 ± 1	0.116 ± 0.02	–4 ± 2	–4 ± 2
150	3.8 ± 0.6	–43.3 ± 0.4	2.0 ± 0.8	–42 ± 1	0.117 ± 0.01	–4 ± 2	–4 ± 2
300	4.6 ± 0.6	–43.8 ± 0.3	2.2 ± 0.7	–41.9 ± 0.8	0.118 ± 0.01	–4 ± 1	–4 ± 1

a All solutions were prepared in phosphate buffer (*I*_buffer_ = 20 mM, pH = 8.0). Data were collected at *T* = 25 °C.

**Table 5:** Same as in Table 4 but for Sucralose

sucralose	I. binding process		II. binding process				
*c* [mM]	*K*_b,1_ × 10^–7^	Δ*G*_b,1_^⊖^ [kJ/mol]	*K*_b,2_ × 10^–7^	Δ*G*_b,2_^⊖^ [kJ/mol]	*n*_2_	Δ*H*_b,2_^⊖^ ×·10^–2^ [kJ/mol]	*T*Δ*S*_b,2_^⊖^ × 10^–2^ [kJ/mol]
0	4 ± 1	–43.4 ± 0.6	2 ± 1	–42 ± 1	0.116 ± 0.02	–4 ± 2	–4 ± 2
150	4.9 ± 0.5	–43.9 ± 0.2	2.5 ± 0.6	–42 ± 1	0.111 ± 0.01	–5.4 ± 0.6	–5.0 ± 0.6
300	11 ± 2	–45.9 ± 0.4	3 ± 1	–42 ± 1	0.106 ± 0.01	–6.7 ± 0.3	–6.2 ± 0.3

**Figure 10:**
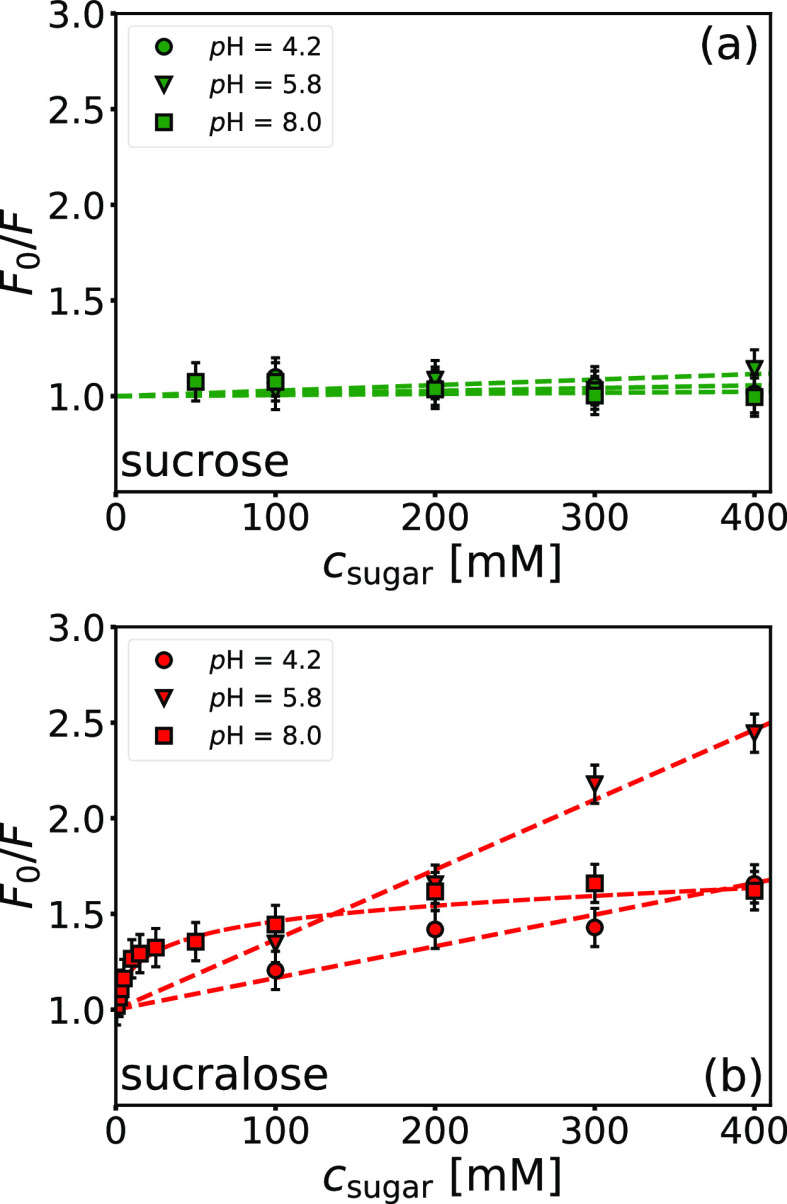
The quenching of BSA fluorescence by sucralose is pH-dependent. Stern–Volmer plots for the quenching of BSA at different (a) sucrose and (b) sucralose concentrations and different pH values. The concentration of BSA was 0.5 μM (λ_ex_ = 280 nm, *T* = 25 °C). All solutions were prepared in buffers with an ionic strength of 100 mM in either acetate buffer (pH = 4.2 and 5.8) or phosphate buffer (pH = 8.0). The data for pH = 4.2 and 5.8 were adopted from our recent work.^32^

The pH-dependent propensity of sucralose toward the BSA surface was partly assessed in our recent work^32^ by fluorescence quenching and molecular dynamics simulations. The analysis focused on pH values around the isoionic point of BSA (pH = 4.2 and 5.8). Here, we extend the analysis to pH = 8.0 (pH ≫ *pI*_BSA_). To elucidate the binding of smaller sugar molecules in the vicinity of hydrophobic regions (hydrophobic amino acid residues) and to be consistent with our previous measurements, the excitation wavelength was set at λ_ex_ = 280 nm (tryptophan, tyrosine, and phenylalanine are excited at this wavelength). The emission spectra of BSA at different sucrose/sucralose concentrations are shown in Figure S15 in the Supporting Information file. The Stern–Volmer plots derived from the emission spectra are shown in Figure 10. Because the presence of both sugars does not cause conformational changes of BSA (see Figure S16 in the Supporting Information file), the quenching of BSA fluorescence is a consequence of direct BSA–sugar interactions with hydrophobic regions of BSA (where the fluorophores are located). By evaluating the quenching at different pH values, the propensity of sucralose toward negatively charged amino acid residues can be elucidated (see discussion in ref (32)). Consequently, a correlation exists between sucralose molecules adhering to glutamic acid (Glu) residues and hydrophobic regions of BSA and depends on the pH value of the medium. At higher pH values, more Glu residues are deprotonated, which increases the tendency of sucralose toward the protein surface, which also leads to more of them quenching the fluorescence of BSA. This is indicated by a very steep dependence of quenching at low sucralose concentrations (*c*_sucralose_ ≲ 15 mM), as shown by the Stern–Volmer plot at pH = 8.0 (Figure 10b). As more sucralose molecules accumulate around the negative regions of the BSA surface, the repulsive interactions between them and the negatively charged −SO_3_^–^ groups of NaPSS are screened, allowing a slightly stronger interaction between the PE and the positive domains of BSA (evident from an increase in *K*_b,1_). The proposed explanation is supported by an increasing binding enthalpy change (Δ*H*_b,2_^⊖^) of the second binding process, as more favorable interactions are formed. This also leads to more BSA molecules adhering to NaPSS, as evidenced by the decreasing binding stoichiometry (the inverse value, 1/*n*_2_, indicates the number of BSA molecules bound per NaPSS) and a more unfavorable entropic contribution as even more configurational entropy of NaPSS is lost during complex formation.

Sucralose exhibits a modulating effect on the protein–PE complexation even in electrostatically dominated systems, which is directly connected with its water-structuring capability around the protein. However, special care should be taken when considering its potential as a stabilizing agent as this strongly depends on the pH of the medium. As such, sucralose is effective in preventing the phase separation, that is, the onset of BSA–NaPSS complex formation at pH ≈ *p*I_BSA_; however, at higher pH values, we showed that it promotes the BSA–NaPSS complexation.

## Conclusions

Complexation between BSA and the synthetic anionic PE (NaPSS) above the isoionic point of BSA was studied by ITC, CD, and absorption and fluorescence emission spectroscopy. The interaction between BSA and NaPSS was evaluated as a function of the mixing order of the components and the presence of co-solutes (salts: NaCl, NaBr, and NaI and sugars: sucrose and sucralose).

It was found that the complexation between BSA and NaPSS at pH = 8.0 (complexation on the “wrong side”) occurs in two closely coupled stages. They correspond to two different binding processes, namely, the formation of intrapolymer complexes and their subsequent association into larger interpolymer complexes. Both binding processes are driven by electrostatic interactions and are possible because positive-charge patches are present on the surface of BSA. It was found that two large positive-charge patches drive the complexation at pH > *pI*_BSA_ and allow complex formation to proceed in a two-stage manner. Both binding processes are accompanied by conformational changes of the protein, but these are more pronounced in the second binding process, that is, the formation of interpolymer complexes.

The presence of salts has the greatest effect on BSA–NaPSS complexation on the “wrong side”. Repulsive interactions between the overall negatively charged macromolecules and attractive interactions between the PE and the positive domains of BSA led to coexistence between SALR repulsive electrostatic interactions mediated by the total ionic strength (*I*_total_) of the solution. Electrostatic screening resulted in a nonmonotonic dependence of binding affinity (apparent binding constants) on *I*_total_. The effect of the increasing salt concentration was different for the two binding processes, probably due to the chain stiffness of NaPSS. The chemical identity of the salt ion also plays an important role, as more chaotropic anions (I^–^, Br^–^) better screen the electrostatic interactions between the macromolecules due to their tendency toward the positively charged regions of the BSA surface.

Complexation is less affected by the presence of sugars. Sucrose does not modulate the interaction between BSA and NaPSS, consistent with the preferential exclusion mechanism, that is, sucrose molecules are excluded from the protein surface and do not influence the electrostatic forces between BSA and NaPSS. Sucralose, on the other hand, shows a tendency toward the BSA surface (preferential binding), especially toward its hydrophobic regions and toward negatively charged parts of the protein (Glu residues). The binding of sucralose molecules to the latter screens the electrostatic repulsion between the net negatively charged macromolecules, which consequently allows a slightly stronger attraction between the positive domains of BSA and the negatively charged PE, leading to a stronger association in the presence of sucralose molecules.

Our findings shed light on the modulatory effect of co-solutes on complexation between simple model systems (BSA, NaPSS) with well-established properties. When considering the modulatory effect of co-solutes on protein–PE complexation, the heterogeneity of the protein surface should be explicitly taken into account. The results contribute to the understanding of complexation between macromolecules in multicomponent systems—information that is crucial for the development of protein drug formulations and other biotechnological applications.

## Supporting Information Available

Raw ITC calorimetric data used to obtain the binding isotherms, model fit of the fluorimetric titration data and comparison of extracted binding constants obtained from ITC and fluorimetry experiments, comparison of the NaPSS-to-BSA and BSA-to-NaPSS titration binding isotherms for co-solute-free solutions, NaPSS-to-BSA binding isotherms at various ionic strengths regulated with NaBr and NaI, absorption spectra at various *r* values for co-solute-free solutions, fluorescence emission spectra at various *r* values for co-solute-free solutions at different temperatures and for systems with added salt (NaCl) and sugar (sucrose, sucralose) at 25 °C, Stern–Volmer plots (and extracted quenching constants) at different temperatures and ionic strengths, influence of the increasing total ionic strength and sugar concentration on the CD spectra, estimates of the BSA secondary structure content extracted from the CD spectra, thermodynamic parameters of the TSIS model for NaPSS-BSA-NaBr and NaPSS-BSA-NaI solutions, dependence of the standard binding entropy change of the second binding process on the ionic strength, electrostatic potential maps of BSA, hydrophobicity map of BSA, and visualization of hydrophobic binding pockets of palmitic acid on HSA (PDF)
