# Supplementary material for: Biomolecular Complexation on the “Wrong Side”: A Case Study of the Influence of Salts and Sugars on the Interactions between Bovine Serum Albumin and Sodium Polystyrene Sulfonate
Source: Biomacromolecules. 2022 Sep 22;23(10):4412–26. doi: 10.1021/acs.biomac.2c00933 (PMC9554918; doi:10.1021/acs.biomac.2c00933)
Supplement: Supplementary file 1 — bm2c00933_si_001.pdf [file bm2c00933_si_001.pdf]

# Supporting Information

# Biomolecular complexation on the "wrong side". A case study of the influence of salts and sugars on the interactions between bovine serum albumin and sodium polystyrene sulfonate.


Matjaž Simončič,[†] Jozef Hritz,[‡,¶] and Miha Lukšič[*,†]

†*Faculty of Chemistry and Chemical Technology, University of Ljubljana, Ljubljana, Slovenia*

‡*Central European Institute of Technology, Masaryk University, Brno, Czechia*

¶*Department of Chemistry, Faculty of Science, Masaryk University, Brno, Czechia*

E-mail: miha.luksic@fkkt.uni-lj.si


## BSA-NaPSS complexation without co-solute present

The ITC signals used to determine the binding isotherm for NaPSS-to-BSA titration (*cf.* Figure 2 in the main text) are shown in Figure S1: Heat effect of mixing BSA and NaPSS and the heat effects of the reference titrations (NaPSS-to-buffer, buffer-to-BSA, and buffer-to-buffer). Phosphate buffer (ionic strenght: 20 mM, $p$H = 8.0) was used. Reference heat effects were subtracted from the BSA-NaPSS signal. We note that at the end of the titration (large $r$ values) $\Delta H$ does not approach zero even after subtracting the reference. This is an intrinsic property of the system under study, and similar was observed by Aberkane et al. (see Figure 2b in Ref. 1). The effect could be attributed to weak non-specific interactions of NaPSS with the saturated complexes. The complexes are thermodinamically unstable and they quickly disintegrate after interaction. Considering that we are evaluating the results comparatively (i.e., in terms of the presence of co-solutes), this effect does not affect the data and the conclusions drawn in the discussion.

In case of BSA-to-NaPSS titration, the concentration of the BSA in the syringe was 200 $\mu$M (approximately 13.3 mg/mL). This concentration is still several times lower than the concentration of BSA in the blood plasma, where it is known that certain fraction of protein molecules can from aggregates.[2] BSA is prone to the formation of dimers at $p$H values around its isoionic point and at high concentrations ($\sim$ 50 mg/mL).[3] Due to the presence of repulsive forces between net negatively charged BSA molecules at $p$H = 8.0, we presume that the concentration of dimers (or larger multimers) in the solution is negligible. In addition, possible heat effects accompanying BSA dissociation upon dilution (BSA-to-buffer titration) were minuscule in comparison to the heat effects of the BSA-to-NaPSS titration. This contribution was nonetheless subtracted from the ITC curve and is therefore accounted for in the extracted thermodynamic parameters.

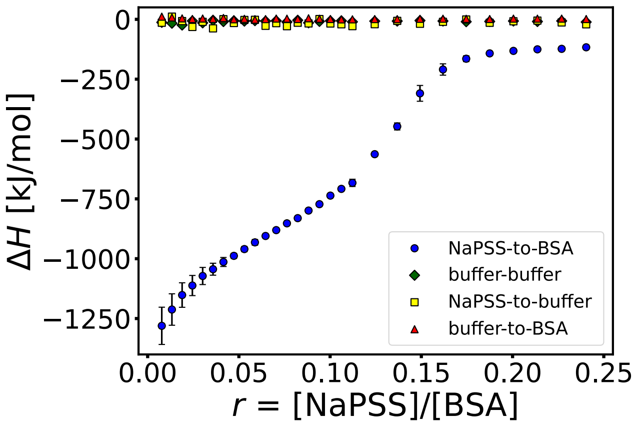


**Figure S1:** The calorimetric signals for the following ITC experiments: NaPSS-to-BSA, NaPSS-to-buffer, buffer-to-BSA, and buffer-to-buffer. All solutions were prepared in the phosphate buffer ($I_{\text{total}} = 20$ mM, $p$H = 8.0) and data were collected at 25 °C.

The complexation between BSA and NaPSS at $p$H = 8.0 occurs above the isoionic point of the protein ($p\text{I}_{\text{BSA}} \approx 4.7$). For this to occur, local attractive interactions must overcome the overall repulsive interactions between the two net negatively charged macromolecules. Electrostatic potential maps at this $p$H and 20 mM ionic strength clearly show the presence of two domains (patches) of positive charge, designated A and B in Figure S2 (left), to which NaPSS can bind with its negatively charged sulfonic groups ($-SO_3^-$). Moreover, under the conditions studied ($I_{\text{total}} = 20$ mM, $p$H = 8.0), complexation proceeds in two stages (*cf.* Figure 2 in the main text) associated with the initial formation of intrapolymer complexes and their subsequent association into larger interpolymer associates as NaPSS concentration increases. For this to be possible, a BSA molecule must be able to facilitate binding to at least two or more binding regions (charge patches), i.e., act as a crosslinker between multiple NaPSS molecules. The presence of the above mentioned binding domains (A and B) supports this statement.

BSA is also known to be a lipophilic molecule. Short-range non-electrostatic interactions between the apolar backbone of NaPSS and the hydrophobic domains of BSA could also be responsible for the complexation at $p\text{H} > p\text{I}_{\text{BSA}}$. The hydrophobicity map of the protein surface is shown in Figure S2 (right). A partially hydrophobic character of BSA can be seen, but no significant hydrophobic domains can be identified, leading us to conclude that short-range non-electrostatic interactions, if present at all, are not the main reason for complexation on the "wrong side" of the isoionic point of BSA nor for the two binding processes at the conditions studied ($I_{\text{total}} = 20$ mM, $p$H = 8.0).

A more global approach to solution thermodynamics to address the binding equilibria from various complementary techniques is in principle more adequate, as different techniques are sensitive to different aspects of the equilibria. The TSIS model was therefore applied also to fluorimetric titration data. Binding isotherm obtained from tryptophan fluorimetry measurements (NaPSS-to-BSA titration) is shown in Figure S3 ($p$H = 8.0, $I_{\text{total}} = 20$ mM). Solid line is the TSIS model fit, and the binding constants for the first and the second process are given in Table S1. For comparison, the binding constants obtained from ITC data are also reported in the table (see Table 1 in the main paper).

**Table S1:** Comparison of the binding constants for both binding processes ($K_{\text{b},1}$, $K_{\text{b},2}$) obtained by fitting the experimental data from the ITC and tryptophan titration experiments.

| | ITC | Fluorimetry |
|---|---|---|
| $K_{\text{b},1}$ | $(4 \pm 1) \cdot 10^7$ | $(7 \pm 2) \cdot 10^7$ |
| $K_{\text{b},2}$ | $(2 \pm 1) \cdot 10^7$ | $(1 \pm 2) \cdot 10^6$ |

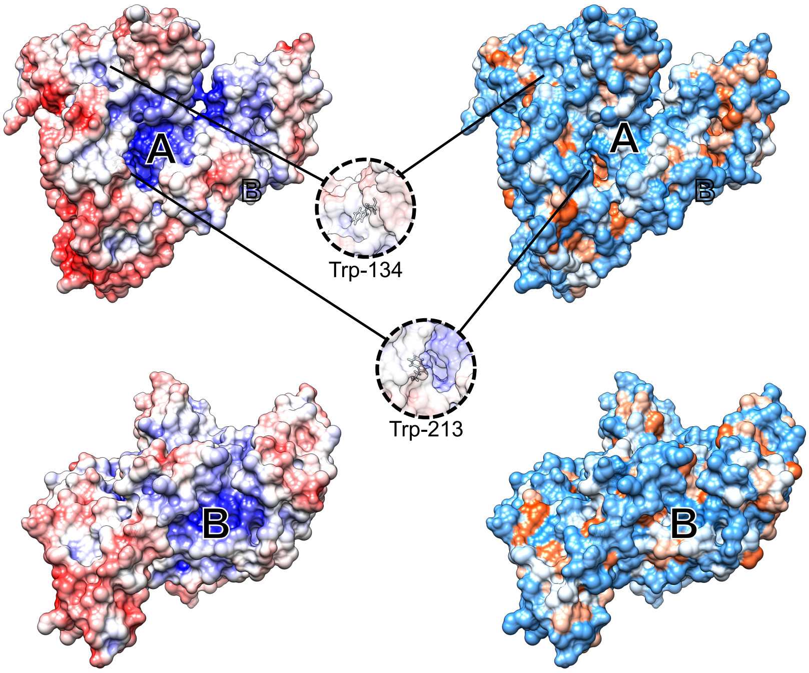


**Figure S2:** *Left*: Electrostatic potential map at the solvent-excluded BSA surface (PDB ID: 4F5S) at $p$H = 8.0, $I_{\mathrm{total}}$ = 20 mM, and 25 °C calculated with PDB2PQR/PARSE[4] and DelPhiPKa.[5] The red color corresponds to $-5\ k_{\mathrm{B}}T/e_0$ and the blue color corresponds to $+5\ k_{\mathrm{B}}T/e_0$ ($k_{\mathrm{B}}$ is Boltzmann constant, $e_0$ is elementary charge, $T$ is absolute temperature). Positively charged patches are labeled A and B. *Right*: hydrophobicity map of BSA plotted using the Kyte-Doolittle hydrophaty index scale[6] (blue: hydrophilic, orange: hydrophobic) and were plotted using Chimera.[7] For details see the main article.

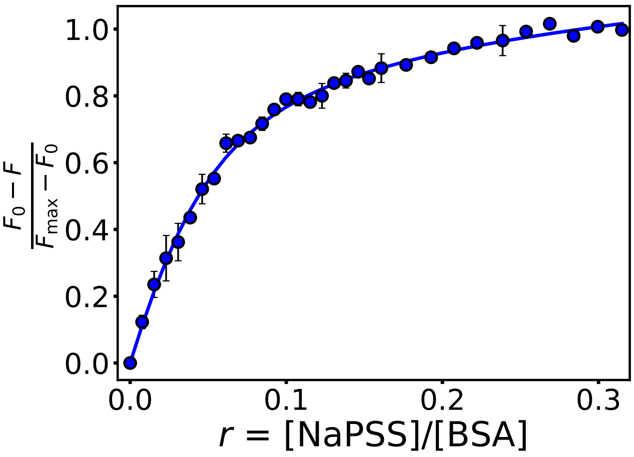


**Figure S3:** The binding isotherm (blue circles) for the NaPSS-to-BSA titration obtained from the fluorimetric measurements ($\lambda_{\mathrm{ex}}$ = 295 nm, recorded at 350 nm). The fit of a TSIS binding model is shown as a solid blue line. All solutions were prepared in phosphate buffer ($I_{\mathrm{total}}$ = 20 mM, $p$H = 8.0) and data were collected at 25 °C. $F_0$ is the fluorescence of the NaPSS-free solution and $F_{\mathrm{max}}$ is the fluorescence at saturation (large $r$).

### NaPSS-to-BSA *vs*. BSA-to-NaPSS titration

Complex formation between BSA and NaPSS depends on the mixing order of the solutions. The binding isotherms for the titration of NaPSS into BSA (NaPSS-to-BSA titration) and BSA into NaPSS (BSA-to-NaPSS titration) are shown in Figure S4. The difference in the binding profiles as well as the extracted thermodynamic parameters (see Table 1 in the main text) can be summarized by different binding mechanisms in excess of BSA or NaPSS (discussed in the main article).

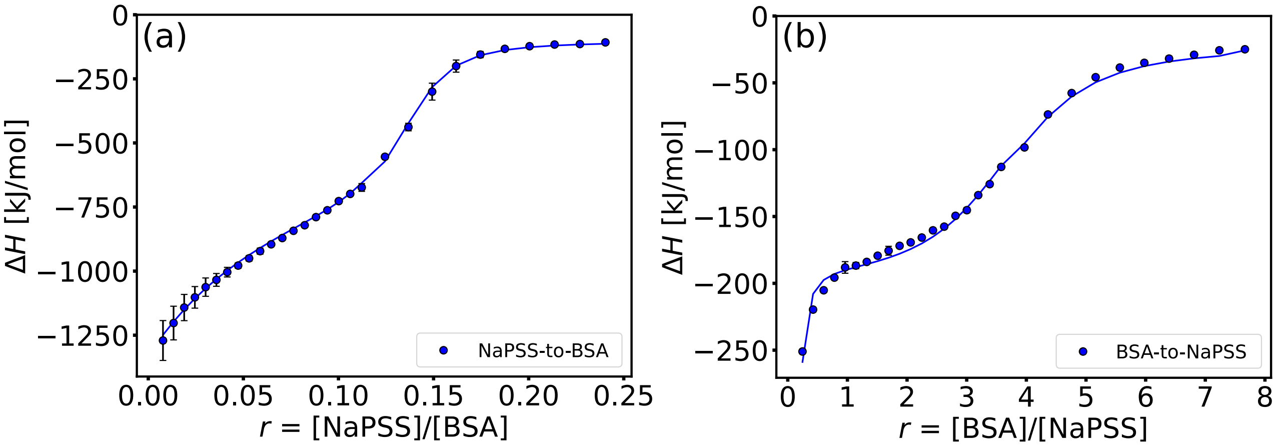


**Figure S4:** Binding isotherms for titration of (a) NaPSS into BSA (NaPSS-to-BSA) and (b) BSA into NaPSS (BSA-to-NaPSS). All solutions were prepared in phosphate buffer ($I_{\text{total}} = 20$ mM, $pH = 8.0$), and data were collected at 25 °C.

### Conformational changes of BSA and binding site determination

As described in the main text (Section *Conformational changes of BSA and binding site determination*) binding of NaPSS to BSA is accompanied by changes in the secondary structure of the protein caused by a reduction in the $\alpha$-helical content and the formation of $\beta$-sheets, as determined from CD spectra using BeStSel software (Table S2). The conformational changes can be correlated with the changes in the molecular environment of the two tryptophan residues of the BSA. As shown in Figure S2, Trp-213 is buried within binding region A and Trp-134 is located in the vicinity of the same binding region near the surface of the protein.

**Table S2:** Estimated secondary structure content (in %) of BSA in the presence of NaPSS at different NaPSS/BSA molar ratios, $r$, as determined by BeStSel software.[8] Data correspond to Figure 3a in the main text. The error was estimated to ±3% from the analysis of two sets of measurements.

| $r$ | $\alpha$-helix | $\beta$-antiparallel sheet | $\beta$-parallel sheet | $\alpha$-turn | others |
|---|---|---|---|---|---|
| 0 | 55 | 1 | 0 | 11 | 33 |
| 0.05 | 56 | 0 | 0 | 12 | 32 |
| 0.10 | 50 | 0 | 0 | 11 | 39 |
| 0.15 | 44 | 3 | 0 | 10 | 43 |
| 0.20 | 45 | 8 | 0 | 11 | 36 |
| 0.25 | 42 | 8 | 0 | 10 | 40 |

The absorption spectra of BSA/NaPSS solutions at different NaPSS/BSA molar ratios, $r$, are shown in Figure S5a. The spectra show a gradual increase in the absorbance of the solution, accompanied by a shift of the absorption maximum to shorter wavelengths with increasing $r$. These changes are primarily related to the increasing absorption of NaPSS in the solution, which becomes evident after subtracting the contributions of the corresponding NaPSS-buffer solutions (Figure S5b). The slight decrease in the absorbance

maximum at 280 nm (inset in Figure S5b) is likely related to changes in the absorption properties of the formed BSA-NaPSS complexes, which could be associated with changes in the molecular environment of tryptophan. However, due to higher sensitivity, the fluorescence measurements provide a more reliable tool for localizing the binding region than does UV-Vis.

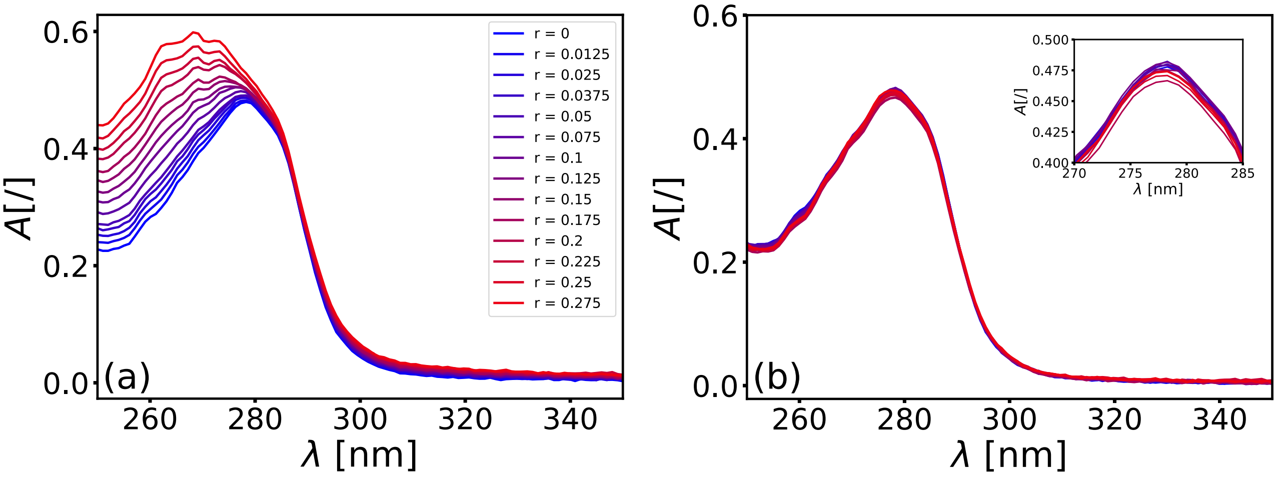


**Figure S5:** Absorption spectra of (a) BSA/NaPSS solutions at different NaPSS/BSA molar ratios, $r$, and (b) the corresponding difference-absorption spectra (subtracted NaPSS-buffer contributions) with changes of the absorbance at 280 nm depicted in the inset. In all cases the concentration of BSA was 9 $\mu$M ($T$ = 25 °C).

The emission spectra of BSA at different NaPSS/BSA molar ratios at 15, 25, and 35 °C are shown in Figure S6. Stern-Volmer plots (Figure S7) and the extracted quenching constants (Table S3) at different temperatures can be used to elucidate the predominant fluorescence quenching mechanism (static or dynamic). Since the interaction between BSA and NaPSS is driven by electrostatic forces, the interactions are weaker at higher temperatures. The Stern-Volmer plots and extracted constants decrease with increasing temperature, which indicates that the predominant quenching mechanism is static.

The dependence of the normalized fluorescence intensity at 350 nm ($T$ = 25 °C) as a function of $r$ (*cf.* Figure 3b in main text) shows that the molecular environment of tryptophans is affected differently in each of the binding processes. Assuming that the binding of NaPSS to the positive patches induces conformational changes near the binding domains, we can deduce that the association between the polyelectrolyte and the protein occurs predominantly on either domain A or B during each binding process.

**Table S3:** Stern-Volmer quenching constants for the first and second binding process ($K_{SV,1}$, $K_{SV,2}$) obtained from the Stern-Volmer plot (2.0 $\mu$M BSA; Figure S7). All solutions were prepared in the phosphate buffer ($p$H = 8.0, $I_{total}$ = 20 mM). Measurements were performed at 15, 25, and 35 °C ($\lambda_{ex}$ = 295 nm).

| $T$ [°C] | $K_{SV,1} \cdot 10^{-3}$ [L/mol] | $K_{SV,2} \cdot 10^{-3}$ [L/mol] |
|---|---|---|
| 15 | 4.2 ± 0.3 | 13.5 ± 0.7 |
| 25 | 3.2 ± 0.2 | 12.1 ± 0.6 |
| 35 | 3.2 ± 0.3 | 9.3 ± 0.5 |

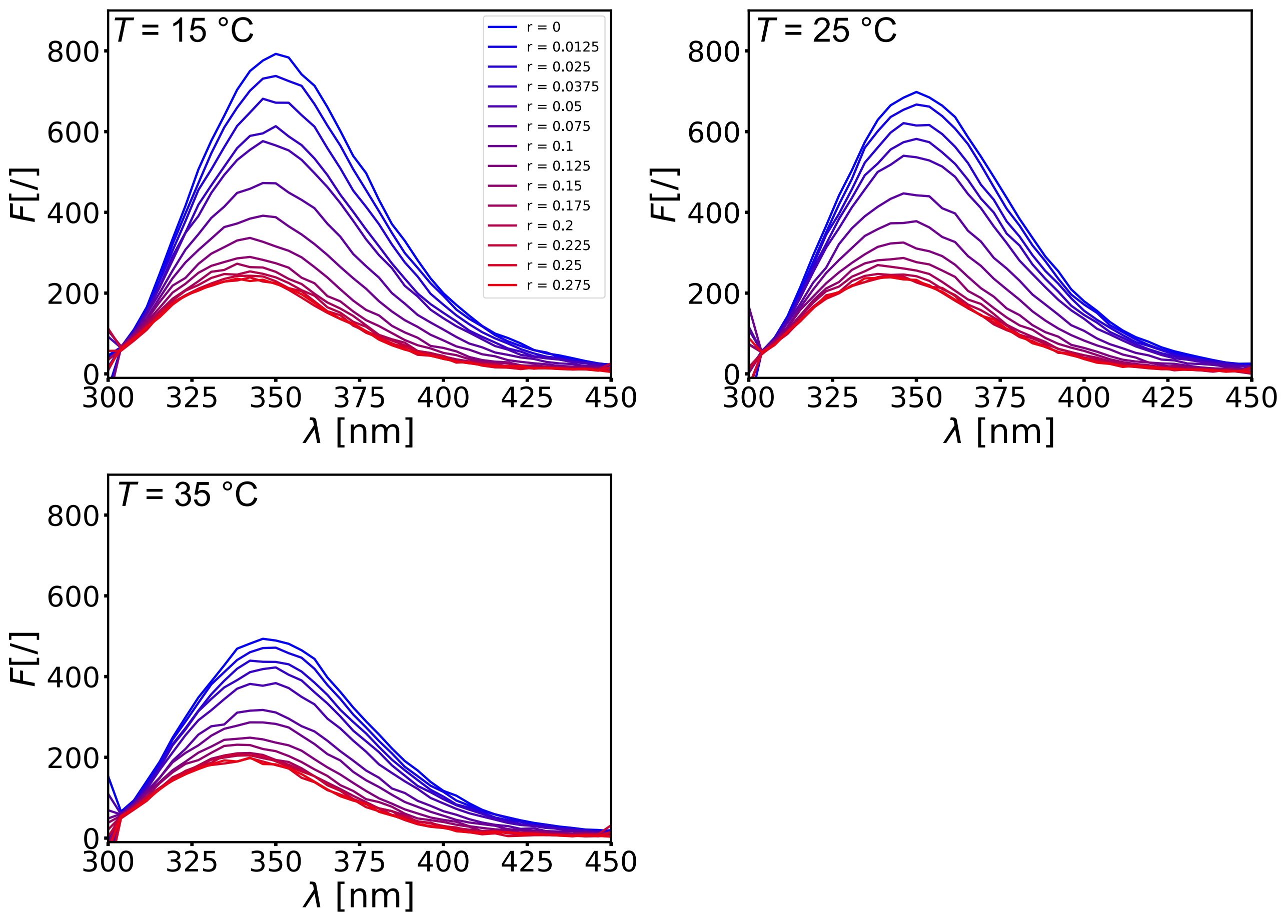


**Figure S6:** Fluorescence emission spectra of 2 $\mu$M BSA at different NaPSS/BSA molar ratios, $r$ ($\lambda_{ex} = 295$ nm) at 15, 25, and 35 °C. Data at 350 nm ($T = 25$ °C) are used for the Stern-Volmer plot (Figure 3b in the main text).

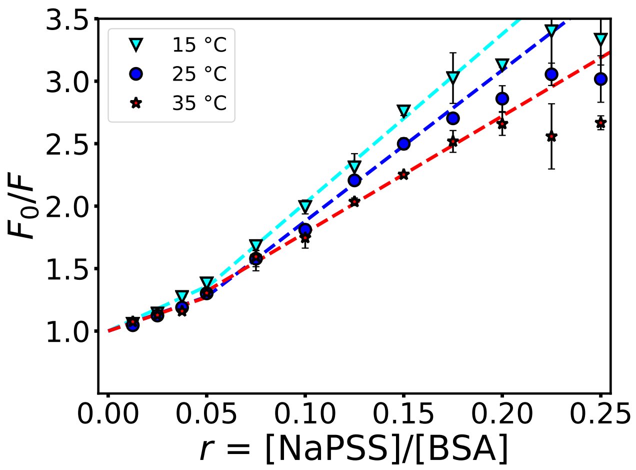


**Figure S7:** Stern-Volmer plots for the quenching of tryptophan fluorescence ($\lambda_{ex} = 295$ nm) at different NaPSS/BSA molar ratios, $r$, and different temperatures, $T$. All solutions were prepared in the phosphate buffer ($p$H = 8.0, $I_{total} = 20$ mM). Data were extracted at 350 nm.

In biomolecular studies purified protein samples are most often used to eliminate potential side effects associated with bound molecules. Since BSA is a fatty acid transporter protein, we evaluate here potential impact of bound fatty acids (FAs) on the complexation between BSA and NaPSS. Binding between BSA (albumins) and FAs usually depends on the chemical properties of FAs (e.g., size and saturation level). For example, if FA has the appropriate size and flexibility, it can fit into one of the hydrophobic pockets of the albumin; otherwise, binding is restricted to the protein surface. In the former case, short-range non-electrostatic interactions between the alkyl chain of FA and the hydrophobic amino acid residues of albumin drive the binding. FAs also have a —COOH group that is deprotonated (negatively charged) under physiological conditions. In such cases, binding occurs at or near the protein surface and is driven by attractive electrostatic interactions between the carboxylate moiety and the positively charged residues of albumin. The multifaceted nature of FAs (size, saturation, etc.) as well as the existence of multiple binding pockets of albumins lead to multiple FA binding sites. Although binding between FAs and BSA was assessed by NMR spectroscopy for BSA-FA complexes with various fatty acid (arachidic, behenic, lignoceric, and hexacosanoic,[9] oleic acid,[10] octanoic acid[11]), crystal structures of BSA-FA complexes, at least to our knowledge, do not exist in the PDB database. However, we can exploit the structural similarity between BSA and human serum albumin (HSA), whose crystal structures with bound FAs are available, e.g., PDB ID 1BJ5 (complex with myristic acid), 1E7F (complex with lauric acid), 1E7H (complex with palmitic acid), etc. To visualize the binding sites of a HSA-FA complex and how they relate to the case of BSA, we selected the HSA complex with palmitic acid and aligned the protein structure of both albumins (PDB ID for BSA was 4FS5). The secondary structures of BSA (orange) and HSA (blue) are shown in Figure S8a using the ribbon representation with a ball-and-stick representation used for palmitic acid (red). By also visualizing the molecular surface (solvent excluded surface area – SESA) of HSA (blue; Figure S8b), we can see that the palmitic acid molecules are embedded in hydrophobic pockets of HSA, which would most likely also be the case with BSA. Interactions between BSA and NaPSS are driven by the electrostatic attraction between negatively charged sulfonic groups of NaPSS ($—SO_3^-$) and positively charged regions (patches) of the BSA and are constrained to the protein surface. Therefore, we can presume that FAs located in the hydrophobic pockets of the BSA would not impact the binding between BSA and NaPSS. Since the $p$H value of the solutions in all our experiments was 8.0, the carboxyl group of FAs would be ionized (negatively charged), leading to attractive electrostatic interactions between the carboxylate moiety and the positively charged regions of BSA. Because nonspecific electrostatic interactions between BSA and NaPSS or FAs are driven by the electrostatic attraction between negatively charged functional groups of the molecules ($—COO^-$ for FAs and $—SO_3^-$ for NaPSS) and positively charged regions of the protein, both could compete for the same binding sites. However, the positive patches on the BSA surface that facilitate binding between BSA and NaPSS under the conditions studied are quite large (domains A and B in Figure S2). Since a NaPSS molecule has multiple neighboring $—SO_3^-$ groups, it would probably displace the bound fatty acid due to the stronger electrostatic attraction. Therefore, we assume that the use of FAs containing BSA would not drastically affect the results obtained in the main article.

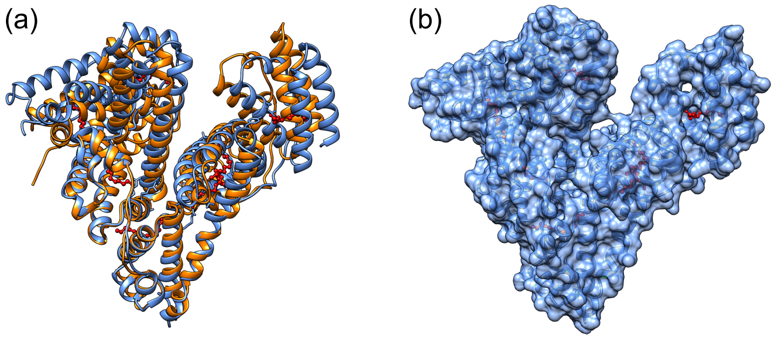


**Figure S8:** (a) The secondary structure of human serum albumin (HSA; blue) and bovine serum albumin (BSA; orange) are presented using ribbons and (b) the molecular surface (solvent excluded surface area – SESA) of HSA (blue). In both cases, palmitic acid molecules are shown using the ball-and-stick representation (red).

# Influence of salts on the BSA-NaPSS complexation

CD spectra at various total ionic strengths, $I_{total}$, and NaPSS/BSA molar rations, $r$, are showin in Figure S9. The conformational changes of BSA upon complexation with NaPSS decrease with increasing ionic strength. Since the secondary structure of BSA consists mostly of $\alpha$-helices, only the $\alpha$-helical content can be considered as an estimate of the conformational changes of BSA (Table S4). It is evident that electrostatic interactions, which are responsible for complex formation, are also the cause of the conformational changes of BSA, since they disappear completely at high ionic strengths. The Stern-Volmer relations shown in Figure S11 as a function of $I_{total}$ (derived from the emission spectra given in Figure S10) confirm this hypothesis, as changes in the molecular environment of tryptophan residues (caused by conformational changes) also decrease nonmonotonically with increasing $I_{total}$ (*cf*. Figure 6 in main text).

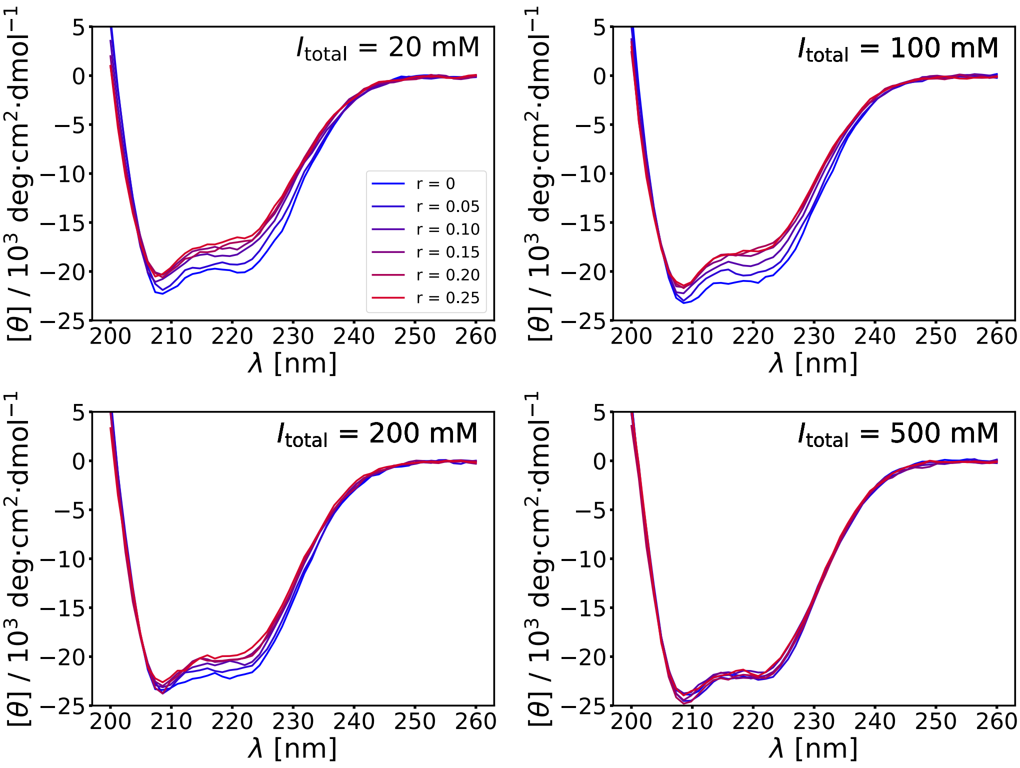


**Figure S9:** CD spectra of 2.5 $\mu$M BSA at different NaPSS/BSA molar ratios and ionic strengths (phosphate buffer at $p$H $= 8.0$). Data were collected at 25 °C.

**Table S4:** Estimated $\alpha$-helical content (in %) of BSA in the presence of NaPSS at different NaPSS/BSA molar ratios, $r$, and ionic strengths, as determined by BeStSel software.[8] Data correspond to Figure S9. The error was estimated to ±3% from the analysis of two sets of measurements.

| $r$ | 20 mM | 100 mM | 200 mM | 500 mM |
|---|---|---|---|---|
| 0 | 55 | 56 | 58 | 63 |
| 0.05 | 56 | 56 | 58 | 65 |
| 0.10 | 50 | 53 | 56 | 66 |
| 0.15 | 44 | 49 | 56 | 62 |
| 0.20 | 45 | 47 | 56 | 65 |
| 0.25 | 42 | 47 | 56 | 65 |

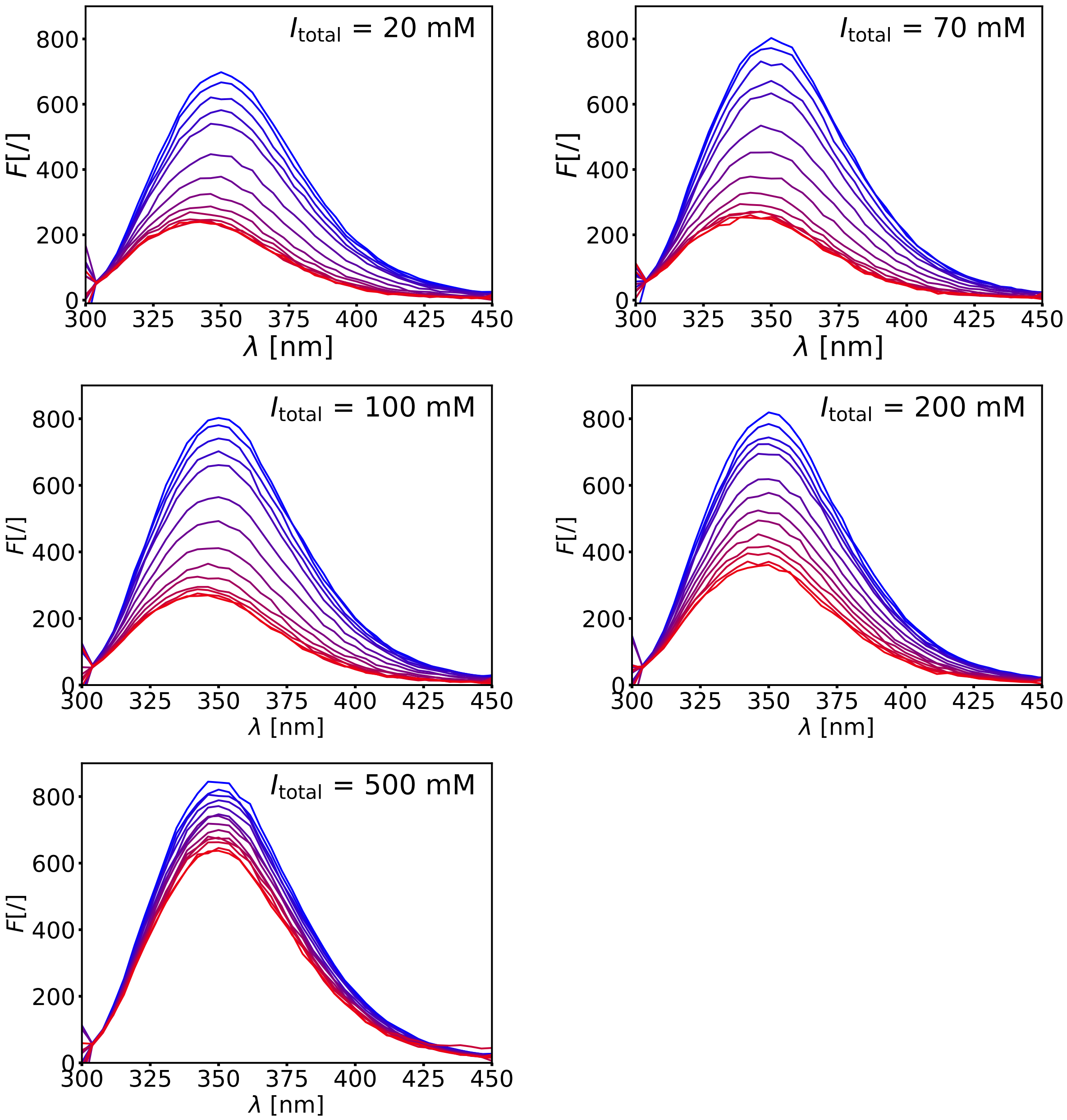


**Figure S10:** Same as in Figure S6 but at different ionic strengths, $I_{\text{total}}$.

Electrostatic potential maps of the BSA surface indicate the presence of two positive domains (charge patches) at all considered ionic strengths (Figure S12). This supports the conclusion that attractive electrostatic interactions between positive domains of BSA and the negatively charged sulfonic groups of NaPSS persists even at high ionic strengths (even up to $I_{\text{total}} = 500$ mM).

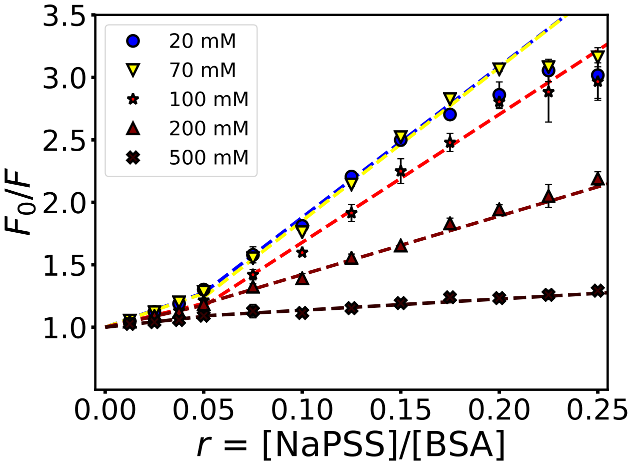


**Figure S11:** Stern-Volmer plots for the quenching of tryptophan fluorescence at different NaPSS/BSA molar ratios, $r$, and different ionic strengths, $I_{\text{total}}$. All solutions were prepared in the phosphate buffer at $p$H $= 8.0$ ($\lambda_{\text{ex}} = 295$ nm, $T = 25$ °C). Data extracted from Figure S10 at 350 nm.

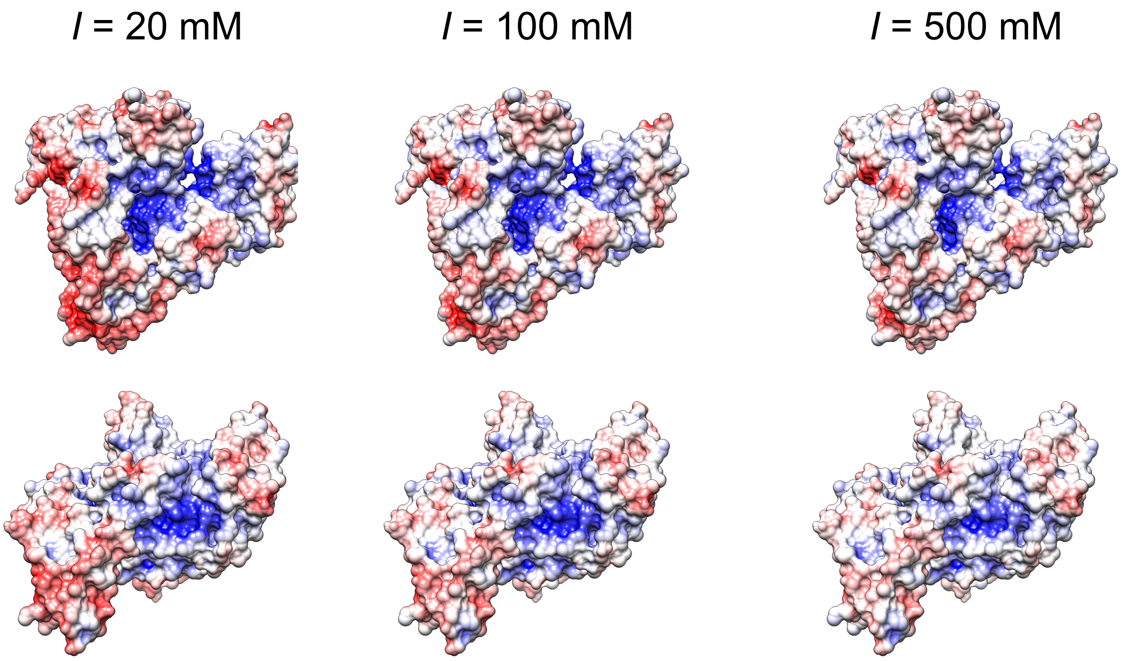


**Figure S12:** Electrostatic potential map at the solvent-excluded BSA surface (PDB ID: 4F5S) at $p$H $= 8.0$ for various ionic strengths ($T = 25$ °C) calculated with PDB2PQR/PARSE[4] and DelPhiPKa[5] (for details see the main article). The red color corresponds to $-5\ k_{\text{B}}T/e_0$ and the blue color corresponds to $+5\ k_{\text{B}}T/e_0$.

The binding isotherms for NaBr and NaI as co-solutes at different $I_{\text{total}}$ can be found in Figure S13 with the TSIS model parameters collected in Tables S5 and S6. The complexation between BSA and NaPSS depends on the chaotropic character of the anion (from more to less chaotropic: $I^- > Br^- > Cl^-$) and is related to the propensity of the anions toward the positively charged patches of the protein. As such, they essentially act as competitors to the sulfonic groups of NaPSS. Consequently, at higher ionic strengths (as well as in the presence of more chaotropic anions), fewer NaPSS molecules are complexed, resulting in fewer polyelectrolyte molecules losing their configurational freedom due to binding. This leads to a less unfavourable entropic contribution to the formation of interpolymer complexes (see Figure S14). Nevertheless, the complexation is still enthalpically driven in all cases (see Tables S5 and S6).

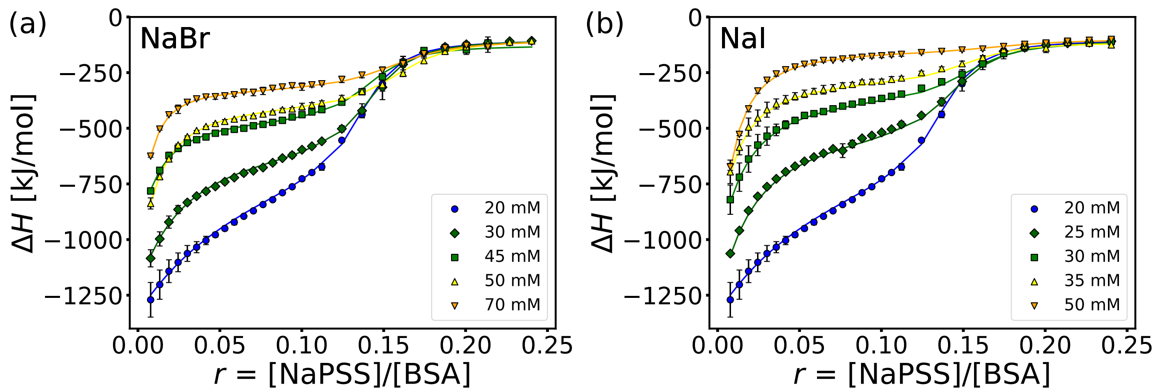


**Figure S13:** Same as Figure 4 in the main text, but for NaBr and NaI as co-solutes.

**Table S5:** Thermodynamic parameters of the first and second binding processes obtained by fitting the TSIS model to the binding isotherm of the NaPSS-to-BSA titration as a function of total ionic strength. All solutions were prepared in phosphate buffer ($I_{buffer} = 20$ mM, $pH = 8.0$) and total ionic strength was adjusted by addition of NaBr (Figure S13a). Data were collected at $T = 25$ °C.

| **NaBr** | **I. binding event** | | **II. binding event** | | | | |
|---|---|---|---|---|---|---|---|
| $I$ [mM] | $K_{b,1} \cdot 10^{-7}$ | $\Delta G^{\ominus}_{b,1}$ [kJ/mol] | $K_{b,2} \cdot 10^{-7}$ | $\Delta G^{\ominus}_{b,2}$ [kJ/mol] | $n_2$ | $\Delta H^{\ominus}_{b,2} \cdot 10^{-2}$ [kJ/mol] | $T\Delta S^{\ominus}_{b,2} \cdot 10^{-2}$ [kJ/mol] |
| 20 | $4 \pm 1$ | $-43.4 \pm 0.6$ | $2.1 \pm 1.2$ | $-42 \pm 1$ | $0.12 \pm 0.02$ | $-4 \pm 2$ | $-4 \pm 2$ |
| 30 | $6.3 \pm 0.7$ | $-44.5 \pm 0.3$ | $1.8 \pm 0.9$ | $-41 \pm 1$ | $0.12 \pm 0.01$ | $-4.7 \pm 0.6$ | $-4.3 \pm 0.6$ |
| 45 | $10 \pm 2$ | $-45.7 \pm 0.5$ | $1.7 \pm 0.6$ | $-41 \pm 1$ | $0.121 \pm 0.010$ | $-3.4 \pm 0.2$ | $-2.9 \pm 0.2$ |
| 50 | $10 \pm 1$ | $-45.6 \pm 0.3$ | $1.4 \pm 0.3$ | $-40.8 \pm 0.5$ | $0.137 \pm 0.005$ | $-3.0 \pm 0.1$ | $-2.5 \pm 0.1$ |
| 70 | $9.4 \pm 0.9$ | $-45.5 \pm 0.2$ | $0.9 \pm 0.1$ | $-39.6 \pm 0.3$ | $0.160 \pm 0.003$ | $-2.16 \pm 0.05$ | $-1.77 \pm 0.05$ |

**Table S6:** Same as Table S5 but for NaI as the co-solute (Figure S13b).

| **NaI** | **I. binding event** | | **II. binding event** | | | | |
|---|---|---|---|---|---|---|---|
| $I$ [mM] | $K_{b,1} \cdot 10^{-7}$ | $\Delta G^{\ominus}_{b,1}$ [kJ/mol] | $K_{b,2} \cdot 10^{-7}$ | $\Delta G^{\ominus}_{b,2}$ [kJ/mol] | $n_2$ | $\Delta H^{\ominus}_{b,2} \cdot 10^{-2}$ [kJ/mol] | $T\Delta S^{\ominus}_{b,2} \cdot 10^{-2}$ [kJ/mol] |
| 20 | $4 \pm 1$ | $-43.4 \pm 0.6$ | $2.1 \pm 1.2$ | $-42 \pm 1$ | $0.12 \pm 0.02$ | $-4 \pm 2$ | $-4 \pm 2$ |
| 25 | $5.9 \pm 0.5$ | $-44.4 \pm 0.2$ | $1.5 \pm 0.4$ | $-40.9 \pm 0.6$ | $0.124 \pm 0.008$ | $-4.0 \pm 0.3$ | $-3.7 \pm 0.3$ |
| 30 | $8 \pm 2$ | $-45.0 \pm 0.7$ | $1.4 \pm 0.9$ | $-41 \pm 2$ | $0.13 \pm 0.02$ | $-2.4 \pm 0.4$ | $-2.0 \pm 0.4$ |
| 35 | $8 \pm 2$ | $-45.1 \pm 0.6$ | $1.2 \pm 0.5$ | $-40 \pm 1$ | $0.132 \pm 0.008$ | $-1.7 \pm 0.2$ | $-1.3 \pm 0.2$ |
| 50 | $7 \pm 4$ | $-45 \pm 1$ | $0.7 \pm 0.5$ | $-39 \pm 2$ | $0.151 \pm 0.009$ | $-0.53 \pm 0.09$ | $-0.14 \pm 0.09$ |

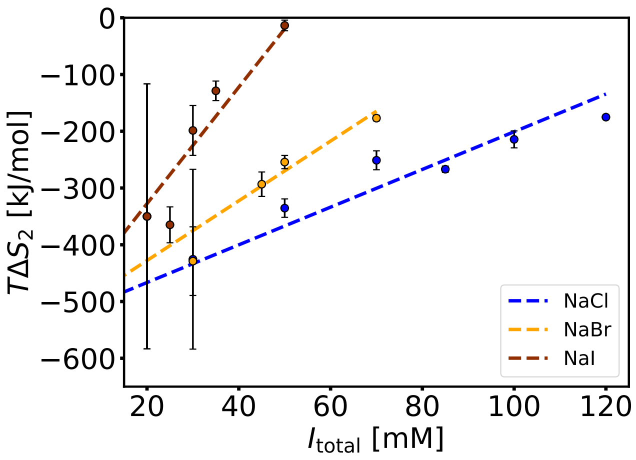


**Figure S14:** Dependence of the standard binding entropy change of the second binding process, $T\Delta S^{\ominus}_{b,2}$, on the total ionic strength, $I_{total}$, at $T = 25$ °C, obtained by the TSIS binding model. The ionic strength of the phosphate buffer ($pH = 8.0$) was adjusted by adding NaCl (blue), NaBr (yellow), or NaI (brown).

# Influence of sugars on BSA-NaPSS complexation

The fluorescence emission spectra of 0.5 $\mu$M BSA in the presence of different sucrose and sucralose concentrations are shown in Figure S15. Since the excitation wavelength was set at 280 nm, other fluorophores besides tryptophan are excited (tyrosine and phenylalanine), and changes in the emission spectrum of BSA at different sugar concentrations can be directly related to changes in the molecular environment of these residues, which are hydrophobic in nature and located near hydrophobic regions of BSA. Since none of the sugars studied cause any conformational changes of BSA (see CD spectra in Figure S16), the changes in the fluorescence signal are associated with BSA-sugar interactions. Since sucrose is preferentially excluded from the BSA surface, no interactions are observed. The same is not true for sucralose, as significant quenching indicates direct protein-sucralose interactions between sucralose and the hydrophobic region of BSA. As can be concluded from our previous work[12] as well as from the $p$H-dependent Stern-Volmer relations (*cf*. Figure 10b in the main text), sucralose molecules also adhere to negatively charged amino acid residues (glutamic acid), making the $p$H-dependent nature of protein-sugar interactions crucial in the treatment of such systems.

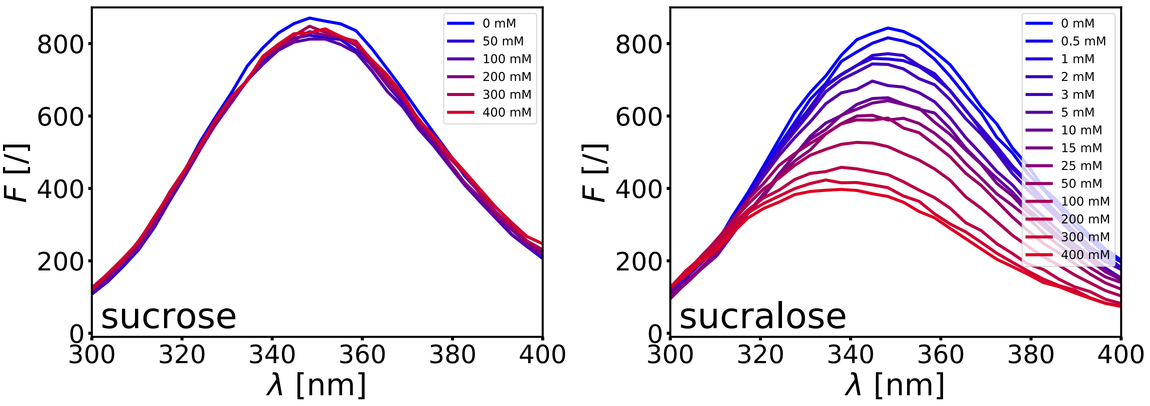


**Figure S15:** Emission spectra of 0.5 $\mu$M BSA with added sugar (sucrose and sucralose) for different sugar concentrations. Solutions were prepared in phosphate buffer with ionic strength of 100 mM at $p\mathrm{H} = 8.0$. Sugar concentrations range from 0 mM (blue) to 400 mM (red). Data correspond to 25 °C ($\lambda_{\mathrm{ex}} = 280$ nm).

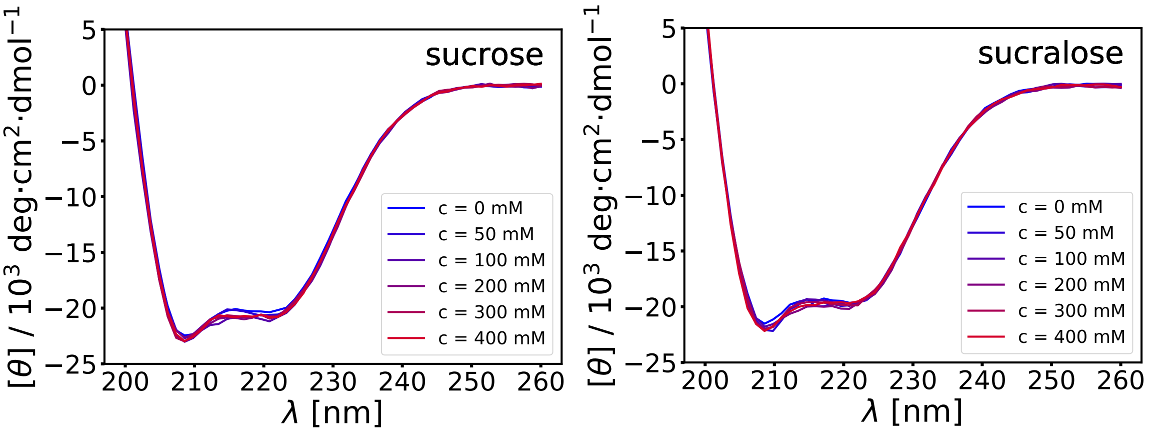


**Figure S16:** CD spectra of 2.5 $\mu$M BSA at different sucrose (left) and sucralose (right) concentrations. All solutions were prepared in the phosphate buffer ($I_{\mathrm{total}} = 20$ mM, $p\mathrm{H} = 8.0$). Data were collected at 25 °C.

# References


(1) Aberkane, L.; Jasniewski, J.; Gaiani, C.; Scher, J.; Sanchez, C. Thermodynamic Characterization of Acacia Gum-$\beta$-Lactoglobulin Complex Coacervation. *Langmuir* **2010**, *26*, 12523–12533.

(2) Peters Jr., T. *All About Albumin: Biochemistry, Genetics, and Medical Applications*; Academic Press, San Diego, 1996.

(3) Barbosa, L. R.; Ortore, M. G.; Spinozzi, F.; Mariani, P.; Bernstorff, S.; Itri, R. The Importance of Protein-Protein Interactions on the pH-Induced Conformational Changes of Bovine Serum Albumin: A Small-Angle X-Ray Scattering Study. *Biophys. J.* **2010**, *98*, 147–157.

(4) Dolinsky, T. J.; Nielsen, J. E.; McCammon, J. A.; Baker, N. A. PDB2PQR: An Automated Pipeline for the Setup of Poisson–Boltzmann Electrostatics Calculations. *Nucleic Acids Res.* **2004**, *32*, W665–W667.

(5) Pahari, S.; Sun, L.; Basu, S.; Alexov, E. DelPhiPKa: Including Salt in the Calculations and Enabling Polar Residues to Titrate. *Proteins* **2018**, *86*, 1277–1283.

(6) Kyte, J.; Doolittle, R. F. A Simple Method for Displaying the Hydropathic Character of a Protein. *J. Mol. Biol.* **1982**, *157*, 105–132.

(7) Pettersen, E. F.; Goddard, T. D.; Huang, C. C.; Couch, G. S.; Greenblatt, D. M.; Meng, E. C.; Ferrin, T. E. UCSF Chimera – A Visualization System for Exploratory Research and Analysis. *J. Comput. Chem.* **2004**, *25*, 1605–1612.

(8) Micsonai, A.; Wien, F.; Kernya, L.; Lee, Y.-H.; Goto, Y.; Réfrégiers, M.; Kardos, J. Accurate Secondary Structure Prediction and Fold Recognition for Circular Dichroism Spectroscopy. *Proc. Natl. Acad. Sci.* **2015**, *112*, E3095–E3103.

(9) Choi, J.-K.; Ho, J.; Curry, S.; Qin, D.; Bittman, R.; Hamilton, J. A. Interactions of Very Long-Chain Saturated Fatty Acids with Serum Albumin. *J. Lipid Res.* **2002**, *43*, 1000–1010.

(10) Hamilton, J. A.; Era, S.; Bhamidipati, S. P.; Reed, R. G. Locations of the Three Primary Binding Sites for Long-Chain Fatty Acids on Bovine Serum Albumin. *Proc. Natl. Acad. Sci.* **1991**, *88*, 2051–2054.

(11) Cistola, D. P.; Small, D. M.; Hamilton, J. A. Carbon 13 NMR Studies of Saturated Fatty Acids Bound to Bovine Serum Albumin. I. The Filling of Individual Fatty Acid Binding Sites. *J. Biol. Chem.* **1987**, *262*, 10971–10979.

(12) Simončič, M.; Lukšič, M. Modulating Role of Co-Solutes in Complexation between Bovine Serum Albumin and Sodium Polystyrene Sulfonate. *Polymers* **2022**, *14*, 1245.
